# Ligand-centered to metal-centered activation of a Rh(iii) photosensitizer revealed by *ab initio* molecular dynamics simulations†

**DOI:** 10.1039/d3sc04381a

**Published:** 2023-11-02

**Authors:** Iria Bolaño Losada, Petter Persson

**Affiliations:** a Division of Computational Chemistry, Department of Chemistry, Lund University Box 124 SE-22100 Lund Sweden petter.persson@teokem.lu.se

## Abstract

Excited state evolution of the rhodium(iii) complex [Rh(iii)(phen)_2_(NH_3_)_2_]^2+^ (phen = 1,10-phenanthroline) has been investigated theoretically to gain a better understanding of light-driven activation of high-energy metal centered states. *Ab initio* molecular dynamics (AIMD) simulations show the significance of asymmetric motion on a multidimensional potential energy landscape around the metal center for activated crossover from triplet ligand centered (^3^LC) to triplet metal centered (^3^MC) states on picosecond timescales. Significant entropic differences arising from the structural distributions of the ^3^LC and ^3^MC states revealed by the simulations are found to favor the forward crossover process. Simulations at different temperatures provide further insight into the interplay between structural and electronic factors governing the ^3^LC → ^3^MC dynamics as a concerted two-electron energy transfer process, and the broader implications for photoinduced generation of high-energy ^3^MC states of interest for emerging photocatalytic applications are outlined.

## Abstract 2

Excited state evolution of the rhodium(iii) complex [Rh(iii)(phen)_2_(NH_3_)_2_]^2+^ (phen = 1,10-phenanthroline) has been investigated theoretically to gain a better understanding of light-driven activation of high-energy metal centered states.

## Graphical abstract

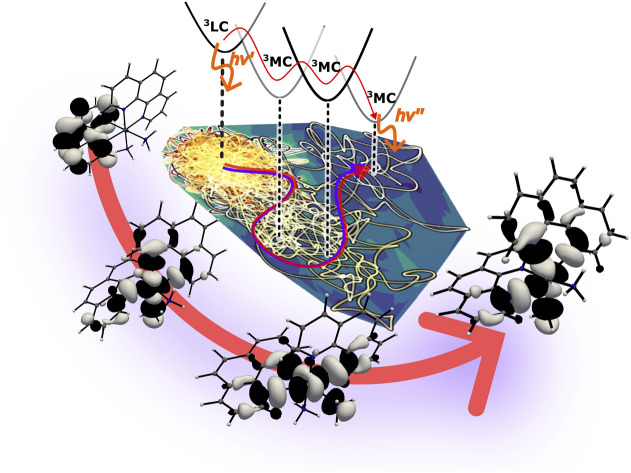

## 1 Introduction

Transition metal complexes that combine efficient light-harvesting with long-lived, high-energy excited states are widely used in molecular photocatalysis and related photochemical applications.^1–3^ Many such photochemical applications have relied on metal-to-ligand charge-transfer (MLCT) excited state schemes, for example with the well-known Ru(ii) polypyridyl type of octahedral d^6^ complexes.^4^ In contrast, involvement of metal centered (MC) excited states has largely been avoided through various ligand design strategies as their population often leads to undesirable excited state energy losses.^3^ Utilization of MC excited states for photochemical applications is, however, emerging as an interesting line of research, with the potential to access a unique set of excited state properties.^5^ This makes it interesting to explore the fundamental photophysical and photochemical properties of suitable prototype complexes in greater detail.^5–8^ Recent progress utilizing transition metal complexes with photoactivated MC states includes several Cr(iii) d^3^ complexes,^9^ some Fe(ii) d^6^ complexes,^10^ as well as a few recent examples of Co(iii) d^6^ complexes.^11–14^ One concern for the photochemical utilization of ^3^MC states is the weak absorption of Laport-forbidden direct dd-excitations. One strategy to overcome this limitation is to activate high-energy ^3^MC states indirectly through energy transfer from another chromophoric unit with better light-harvesting capabilities.^8^ This can be achieved as an intramolecular activation from an initially excited CT or ligand centered (LC) state. Another challenge with the involvement of MC states in d^6^ complexes is the population of anti-bonding e_g_ orbitals that frequently causes substantial excited state energy losses, associated with weakened metal–ligand (M–L) bonds.^15,16^ Activated crossover into high-energy ^3^MC states following initial LC excitation is, however, known from studies of prototype Rh(iii) complexes, as evidenced by experimental observations of dual photoluminescence (PL) from both the ^3^LC and ^3^MC states, as well as photochemical quenching experiments involving the ^3^MC state.^17–26^ The effect on the relative energy between the ^3^LC and ^3^MC and its impact on the state-to-state crossover efficiency was studied experimentally for a series of tris-phenanthroline derivatives, with the general formula [Rh(iii)(phen)_2_XY]^*n*+^ (phen = 1,10-phenanthroline), by tuning the ligand field strength.^19^

Quantum chemical simulations offer significant opportunities to gain detailed insight into several different aspects of the excited state evolution of transition metal complexes. A variety of many-state quantum dynamical simulation methods have provided important insights into the ultrafast dynamics associated with initial relaxation processes within a dense manifold of excited states.^27–35^ Some simulations have also covered the explicit interplay between excited state deactivation processes and solvent relaxation and reorganization processes.^36^

In this work, we investigate the ^3^LC → ^3^MC state crossover activation in the prototype Rh(iii) phenanthroline complex, [Rh(phen)_2_(NH_3_)_2_]^3+^, using *ab initio* molecular dynamics (AIMD) simulations conducted at the density functional theory (DFT) level. The [Rh(phen)_2_(NH_3_)_2_]^3+^ complex provides a good prototype system to investigate such ^3^LC → ^3^MC crossover dynamics computationally as experimental observations of dual emission in the 170–200 K temperature range point to a favorable ^3^LC–^3^MC balance, similar to what has also been reported for the [Rh(iii)(phen)_3_]^3+^ complex.^19,24,37,38^ We focus on intramolecular aspects of activated ^3^LC → ^3^MC crossover dynamics on picosecond timescales, *i.e.* excited state dynamics taking place on the lowest excited triplet potential energy surface following any initial ultrafast ISC and IC processes as well as initial local relaxation of the initially populated ^3^LC state. This provides a largely complementary set of computational challenges compared to many recent studies focused on the earliest ultrafast multi-state relaxation processes, and places a greater focus on accounting for large-scale structural distortions on a complex multidimensional molecular energy landscape on timescales that can extend to tens of picoseconds and longer.

## 2 Computational details

Full geometry optimizations of structurally distinct relaxed ^3^LC and ^3^MC states in the [Rh(phen)_2_(NH_3_)_2_]^3+^ complex were performed at the unrestricted DFT (uDFT) level of theory using the B3LYP* hybrid functional with reduced 15% Hartree–Fock exchange (HF_ex_) and the 6-311G(d,f) basis set with the Stuttgart–Dresden SDD effective core potential for the central Rh atom.^39–42^ An implicit Polarizable Continuum Model (PCM) was applied to account for a water solvent environment.^43^ The nature of the various states was confirmed by frequency analysis. All calculations were conducted using the Gaussian09 software.^44^

Optimized structures at the B3LYP*(15% HF_ex_)/6-311G(d,f)/(water) in the ^3^MC and ^3^LC excited states were chosen as starting geometries for subsequent AIMD calculations using the atom centered density matrix propagation (ADMP) model.^45^ The size of the basis set was reduced to 3-21G in order to reduce the computational costs, and motivated by test calculations indicating only minor differences for the calculated energy differences between the relaxed ^3^LC and ^3^MC states according to a comparison with the larger 6-311G(d,f) basis set. Explicit solvent structure and dynamics were neglected in this study focusing mainly on intramolecular rearrangement aspects, and an overall solvent response was approximated with the PCM model. The temperature dependence of the dynamics was investigated at constant selected temperatures (170 K, 200 K, 298 K, and 330 K) imposed by a standard thermostat. A minimum energy path was calculated as a complementary way to track the ^3^MLCT → ^3^MC conversion (details in ESI†).

## 3 Results and discussion

### 3.1 Triplet potential energy surface

**Fig. 1:**
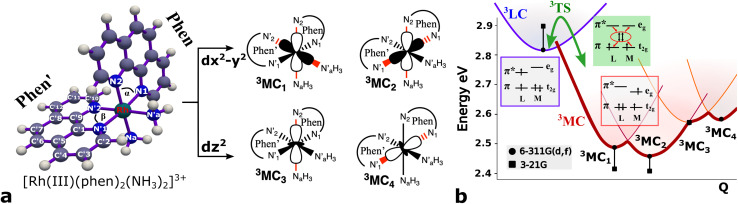
Chemical structure and triplet excited state properties of the [Rh(iii)(phen)_2_(NH_3_)_2_]^3+^ complex. (a) Chemical structure with key labels and e_g_ metal orbital orientations in [Rh(iii)(phen)_2_(NH_3_)_2_]^3+^ metal complex. (b) Triplet excited state energies (black) for the ^3^LC and ^3^MC states. The energy is referenced to the ground state energy in eV. Dots designate B3LYP*/6-311G(d,f)/PCM(water), and squares designate B3LYP*/3-21G/PCM(water) results. The calculated energies are combined with a schematic description of the conversion between ^3^LC and ^3^MC states.

Several local triplet state minima were identified for the [Rh(iii)(phen)_2_(NH_3_)_2_]^3+^ complex from a first exploration of the triplet PES by means of unconstrained geometry optimizations and single point quantum chemical calculations. A single ^3^LC state minimum, identified as a local excitation on one of the phenanthroline ligands (designated as phen′), as well as a set of four non-degenerate local minima of ^3^MC nature were identified by geometry optimizations. Different orientations of the e_g_ orbitals relative to the M–L bonds allow for up to six different ^3^MC states associated with Jahn–Teller distortions along different M–L axes. The *C*_2_ rotation axis, characteristic of the *C*_2_ point group to which [Rh(iii)(phen)_2_(NH_3_)_2_]^3+^ complex belongs more specifically, reduces the number of unique ^3^MC states to four. A graphical representation of the e_g_ orbital orientations along different M–L bonds, together with the names for the corresponding states adopted in this report (^3^MC_1_, ^3^MC_2_, ^3^MC_3_ and ^3^MC_4_), are described in Fig. 1. Additionally, it can be noted that the more distorted octahedral coordination in the heteroleptic complex breaks the orbital degeneracy in the e_g_ orbital set more strongly compared to the homoleptic parent complex [Rh(iii)(phen)_3_]^3+^. This potentially increases the energy offset between the distinct ^3^MC states. Indeed, different emission band broadening characteristics for the two Rh complexes of ∼40 cm^−1^ in acetonitrile at 297 K for [Rh(iii)(phen)_3_]^3+^ and ∼60 cm^−1^ in a water/ethylene mixture at 170 K for [Rh(iii)(phen)_2_(NH_3_)_2_]^3+^ provides a likely experimental indication of such differences in the MC state PES landscapes.^19,38^

The M–L bonds of the relaxed ^3^MC state geometries show bond elongations and contractions with respect to the ^3^LC, similar to what has been reported for several ruthenium polypyridine complexes.^16,46–48^ The Jahn–Teller distorted ^3^MC states can thus be identified to involve a single axis for e_g_-d_*z*^2^_ type orbitals, or an equatorial mode involving two orthogonal axes for e_g_-d_*x*^2^−*y*^2^_ type orbitals. The ^3^MC_1_, ^3^MC_2_, ^3^MC_3_ and ^3^MC_4_ states were identified at the B3LYP*/6-311G(d,f) computational level, while only the first three ^3^MC states were found at the lower level of theory B3LYP*/3-21G (energies shown in Fig. 1b). Reasonable reliability of the calculations employing the 3-21G basis set was, however, validated against the results for the larger 6-311G(d,f) basis set in terms of the relative state energy trends agreeing to within 0.05 eV, and average M–L bond lengths agreeing to within 0.004 Å (details in Fig. SI.1, Tables SI.2 and SI.3†). Furthermore, the relative energy ordering among the ^3^MC and ^3^LC states is conserved with the smaller basis set. The calculations predict ^3^MC_2_ to be the lowest energy ^3^MC state, and thus the most likely populated states in the triplet energy surface according to Kasha's rule (2.46 eV and 2.41 eV above the singlet ground state (^1^GS) for the B3LYP*/6-311G(d,f) and B3LYP*/3-21G levels of theory, respectively). Additionally, an almost isoenergetic second equatorial distorted ^3^MC_1_ state was found to be higher in energy by only 0.03 eV at the B3LYP*/6-311G(d,f) and 0.01 eV at the B3LYP*/3-21G levels of theory, respectively. Both axial distorted states, ^3^MC_3_ and ^3^MC_4_, were calculated to be ∼0.1 eV higher compared to the equatorial distorted states. Gibbs free energies also support the equatorial distortion as the most favorable compared to the axial distortion (Table SI.3†). From the first principles calculations, population of either the ^3^MC_2_ or the ^3^MC_1_ state can thus be expected as a result of excited state relaxation, although no information can be immediately extracted regarding the depopulation mechanism of the initially populated ^3^LC state. AIMD simulations were therefore initiated for the [Rh(iii)(phen)_2_(NH_3_)_2_]^3+^ complex, with the aim to reveal the crossover dynamics as well as further properties associated with the population dynamics of thermally accessible states.

### 3.2 ^3^LC dynamics

To better understand the triplet state dynamics in [Rh(iii)(phen)_2_(NH_3_)_2_]^3+^ after initial optical excitation and intersystem crossing into a ^3^LC excited state, we initially performed AIMD calculations at 170 K, 200 K, and 298 K.

A detailed analysis of the spin density distributions on the different molecular fragments from 7.5 ps simulations conducted at 170 K, 200 K, and 298 K only shows significant spin density values for one of the two phenanthroline ligands (labelled phen′ in Fig. 1a) with average spin density values of 1.99 (full spin density progression in Fig. SI.5†). The large spin density on the phen′ ligand indicates that the lowest energy ^3^LC states is characterized by a localized electron excitation on that particular ligand. Any ligand-to-ligand excitation transfer is discarded within the studied temperatures and time range since the spin density values remain essentially negligible on all the other ligands. The spin density localised on the Rh metal of barely ∼0.01 also contradicts conversion to, or strong M–L mixing with, any ^3^MC state in the simulation.^19,38^ This spin density distribution on the phen′ fragment was further analyzed over the simulation (details in ESI†). The spin density is mainly delocalised over the inner part of the phenanthroline frame. In particular the 
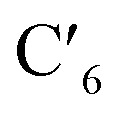
 and 
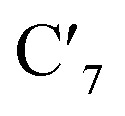
 carbons, as numbered in Fig. 1a, accumulates larger spin density (∼2.0) during the 7.5 ps simulation. The electronic structure in the ^3^LC state also correlates with stiff M–L bonds with all six M–L bond distances ∼ 2.1 Å, and with fluctuations of merely 0.05 Å at 298 K, 0.04 Å at 200 K and 0.03 Å at 170 K (details in Fig. SI.3†). Both structural and electronic properties are thus consistent with triplet dynamics constrained to a localized ^3^LC excited state that only displays small motion in close vicinity of the ^3^LC structural minimum.

### 3.3 ^3^MC dynamics

Static uDFT calculations predicted the presence of four distinct ^3^MC states (^3^M_1_, ^3^MC_2_, ^3^MC_3_ and ^3^MC_4_), but the dynamic distribution and conversions between these states are unknown. Therefore, AIMD simulations were also considered on the MC triplet PES at 170 K, 200 K, and 298 K with the aim to investigate transitions between the different local ^3^MC states, as well as the multi-dimensionality of the global ^3^MC PES.

**Fig. 2:**
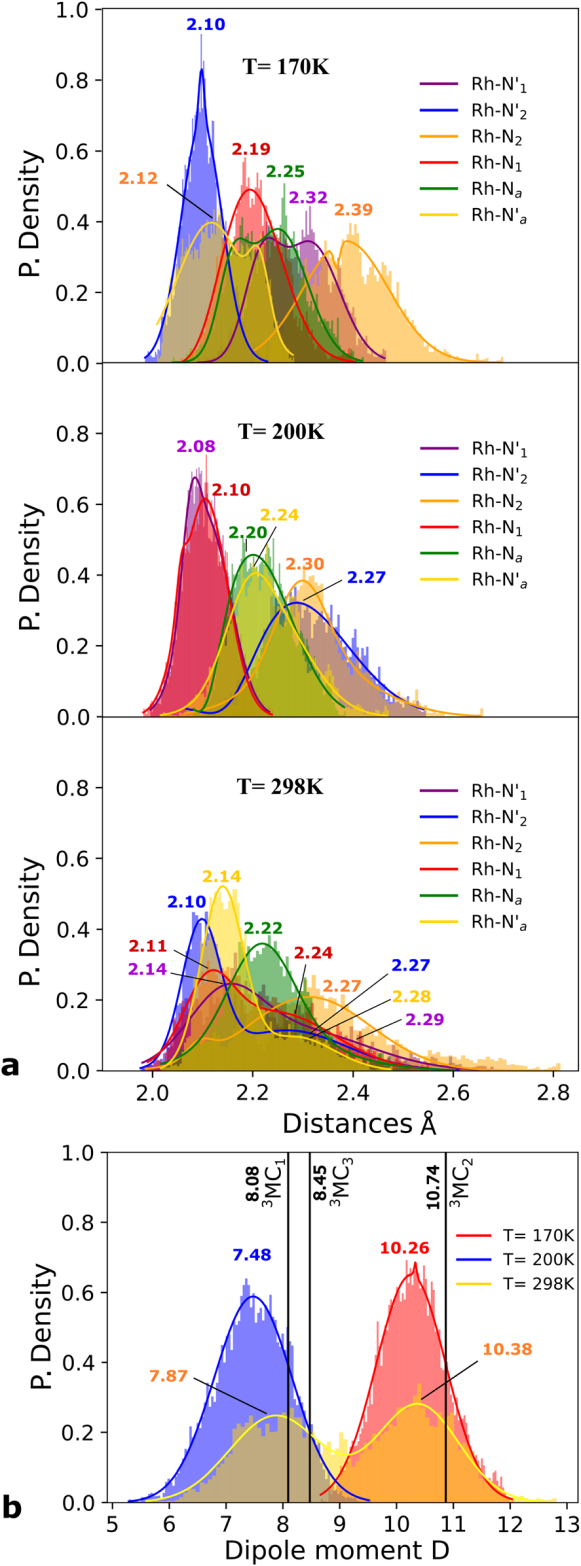
Probability distributions (P. density) (a) bond distance in Å for the six metal–ligand bonds in the ^3^MC state of the [Rh(iii)(phen)_2_(NH_3_)_2_]^3+^ complex: 
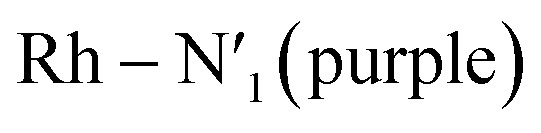
 and 
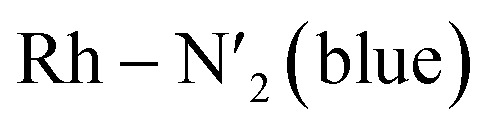
 in phen′, Rh – N_1_ (red) and Rh – N_2_ (orange) in phen, and 
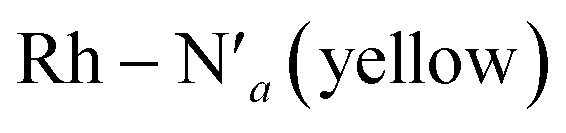
 and Rh – N_*a*_ (green) during a 7.5 ps AIMD sampling at 170 K, 200 K, and 298 K. (b) Dipole moment in D for the ^3^MC state in the [Rh(iii)(phen)_2_(NH_3_)_2_]^3+^ complex for 7.5 ps AIMD sampling at (red) 170 K, (blue) 200 K, (yellow) 298 K. Vertical black lines mark the calculated dipole moment in D at the uDFT minimized structures for the ^3^M_1_, ^3^MC_2_, and ^3^MC_3_ excited states.

M–L bond distance distribution plots are analysed in Fig. 2a for the selected temperatures. The M–L bond distance distributions for the 200 K simulation show sharp peaks for all of the six bonds that are significantly displaced to longer bond distances compared with the ^3^LC state bond distributions. Three sets of bond distributions with similar mean distances and peak broadening that reveal similar bond characteristics can be identified (
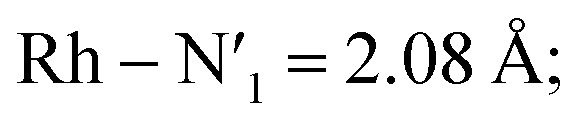
 Rh – N_1_ = 2.10 Å; 
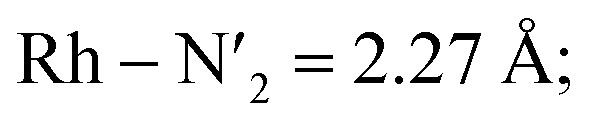
 Rh – N_2_ = 2.30 Å). Such structural asymmetry is consistent with general expectations for Jahn–Teller distortions due to an e_g_ metal d-orbital population. Similar bond distortions of up to ∼0.2 Å in a ^3^MLCT → ^3^MC transition with equatorial distortion in [Ru(ii)(bqp)_3_]^2+^ complex has, for example, also been reported at the uDFT level.^48^ All the bond elongations and contractions at 200 K suggest the population of a single d_*x*^2^−*y*^2^_ d-type-orbital, and the M–L bond values also correlate well with the calculated distances in the fully relaxed ^3^MC_1_ excited state.

A clearly different set of M–L bond distance distribution profiles (top Fig. 2a) at the temperature of 170 K compared to what was seen for 200 K provides a first indication that another distinctive ^3^MC excited state is populated in this simulation. Two bond distributions with average distances of ∼2.1 Å (blue and orange peaks) are shorter than the remaining four coordination sites in agreement with an e_g_-d_*x*^2^−*y*^2^_ orbital assignment characteristic of the ^3^MC_2_ state (Fig. 1a). Other prominent structural changes due to the reorientation of the d-orbital lobes towards both chelation sites in the phen ligand is the reduction of the bite angle *α* by 8 degrees, if compared with the ^3^LC geometry (Fig. SI.7†). The geometry distortion in the lowest energy ^3^MC_2_ state also affects the electronic properties significantly, as evidenced from the clear fluctuation in the calculated total dipole moment of the molecule for the simulations conduced at different temperatures according to Fig. 2a. This shows an increase of the dipole moment from 7.48 Debye (D) (blue peak) in the ^3^MC_1_ state (200 K simulation) to 10.26 D (red peak) corresponding to the ^3^MC_2_ state (170 K simulation). Both ^3^MC_1_ and ^3^MC_2_ calculated dipole moments by uDFT minimization calculations (8.08 D and 10.74 D) reinforce the general state assignments, and the electronic identity of each ^3^MC state. Larger peak broadening additionally suggest that the ^3^MC_1_ is able to move over a larger accessible volume in the multidimensional PES compared to the ^3^MC_2_ state.

Finally, we consider a simulation of the ^3^MC dynamics at 298 K. At this higher temperature, the M–L bond distributions access a wide range of distances during the simulation, and with more than one distribution peak. Furthermore, the dipole moment histogram at 298 K shows a bimodal distribution characteristic of alternating population of two relatively distinct local electronic states (yellow distribution in Fig. 2b).

**Fig. 3:**
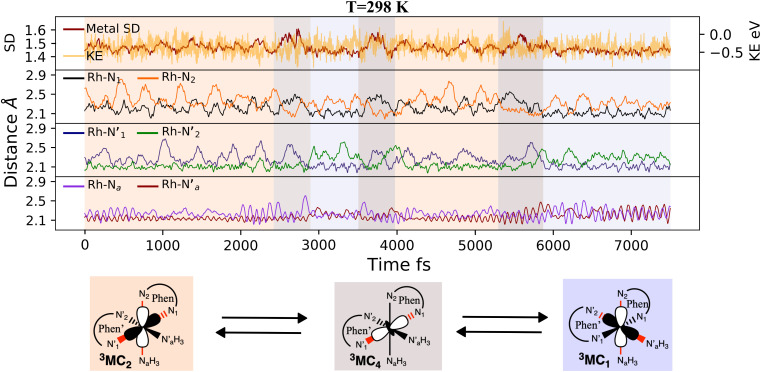
^3^MC AIMD trajectories for 7500 fs at 298 K. (top panel) metal spin density (SD) in red and kinetic energy (KE in eV) fluctuations in yellow. (Lower panels) metal–ligand bond distances (in Å) for phen (black and yellow), phen′ (blue and green), and the two amine groups (purple and red).

The AIMD structural data for the 298 K simulation also indicates population of at least two ^3^MC states with populated d_*x*^2^−*y*^2^_ orbital, but different orbital orientation. For further understanding of the dynamics between different local ^3^MC states, the trajectories of the six bonds were classified according to the relative bond distance ordering in Fig. 3. A remarkable transition between bond distances occurs after 2500 fs during a total transition time of ∼400 fs (grey frame). Finally, the 2900–3500 fs time interval (blue frame) shows bond distances that fully correlate with the ^3^MC_1_ excited state. Repetitive transition pattern between ^3^MC_1_ and ^3^MC_2_ states observed in the continuation time sampling within the 7500 fs, alludes to the established equilibrium between ^3^MC_1_ and ^3^MC_2_ states. The state transition, which also coincides with a discontinuity in the metal spin density, displays simultaneous symmetrical Rh – N_1_ and 
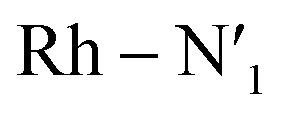
 and asymmetrical N_*a*_ and 
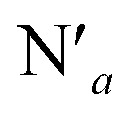
 bond stretching. Electronic and structural attributes of the identified intermediate state coincides with the uDFT relaxed structure referred to as ^3^MC_4_, and distinguished by the characteristic axial elongation associated with electron population of one d_*z*^2^_ orbital. Therefore the dynamical equilibrium between the two equatorial states and lower lying ^3^MC states was revealed to implicate transient population of at least one higher energy ^3^MC states. Based on the observed transitions, the rate constant *k*_IC_ for the ^3^MC_1_ → ^3^MC_2_ IC can be estimated to be 2.5 × 10^12^ s^−1^ at room temperature.

### 3.4 ^3^LC → ^3^MC crossover dynamics

Here, we consider longer MD simulations starting from the ^3^LC state and performed with the aim to investigate the crossover dynamics from ^3^LC to the ^3^MC state manifold. The uncertainty of the crossover timescale, together with the large computational requirements for long AIMD simulations encouraged us to sample moderately higher temperatures of 298 K and 330 K.

**Fig. 4:**
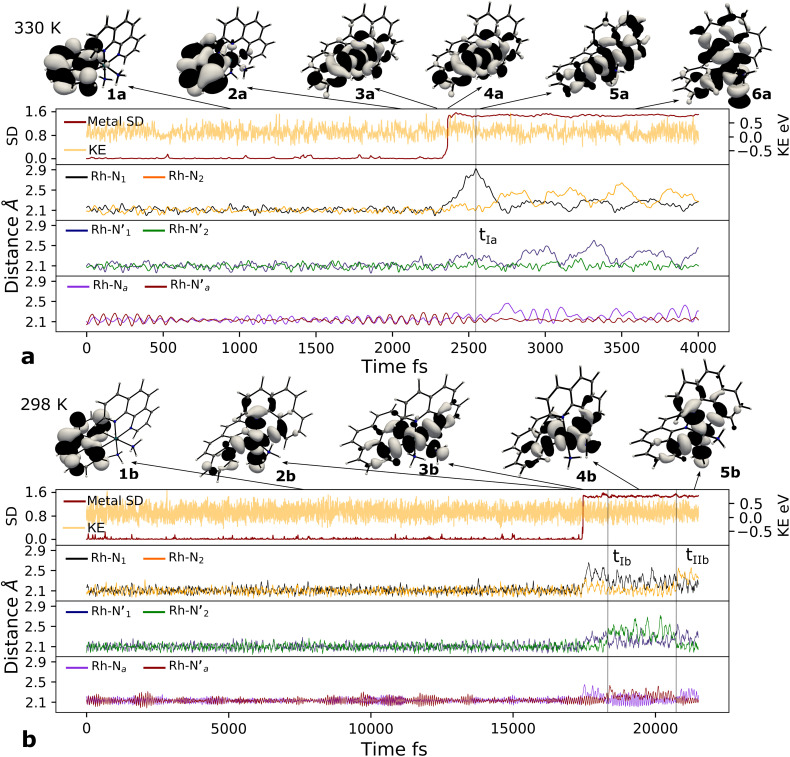
Metal–ligand bond stretching of [Rh(iii)(phen)_2_(NH_3_)_2_]^3+^ (Rh – N_1_ in black, Rh – N_2_ in yellow, 
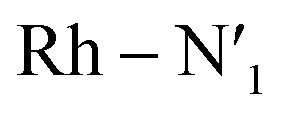
 in blue, 
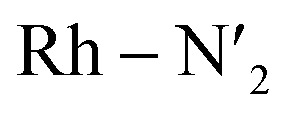
 in green, Rh – N_*a*_ in purple, 
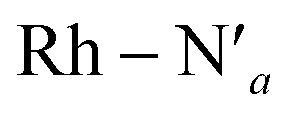
 in red) before and after ^3^LC → ^3^MC crossover reaction, metal spin density (SD) and kinetic energy (KE) fluctuation at temperatures of (a) 330 K, (b) 298 K. ^α^SOMO orbitals are plotted for several snapshots along the simulation.

First, an AIMD simulation at 330 K shown in the top panel of Fig. 4a showed an abrupt increase in the metal spin density after ∼2300 fs. During the transition, the metal spin density increases from 0.1 to 1.4, indicating that the ^3^MC is populated within a couple of picoseconds. Further insight into the electronic structure rearrangement taking place during the crossover can be extracted from consideration of the singly occupied and unoccupied frontier molecular orbitals participating in the electronic transition. The ^α^SOMO orbital represented as 1a in Fig. 4a shows the predominant π ligand orbital characteristic of the ^3^LC, but with metal-d contributions to the orbital escalating as the system approaches the transition state. Just before the electronic structure redistribution, only small-scale structural changes in the M–L bond distances (with constant ∼ 2.1 Å distances) are registered, mainly in the Rh – N_1_ bond reaching a distance of 2.23 Å right before the transition occurs. ^α^SOMO orbital (3a in Fig. 4a) at the discussed simulation time already suggests some atomic contribution from a metal d-orbital. The Rh – N_1_ bond stretching is, however, only emphasized after ∼200 fs, indicating that there is a sub-picosecond geometry equilibration period after the electronic crossover to the ^3^MC state. The Rh – NH_3_ and Rh – N_2_ bond distances are barely distorted during this equilibration interval of 200 fs, which is indicative of an axial Jahn–Teller distortion of the ^3^MC_4_ state. The first deactivation step of the ^3^LC excited state to a higher energy ^3^MC state is also confirmed by the nature of the ^α^SOMO orbital 4a with a characteristic d_*z*^2^_ orbital lobe orientation along the 
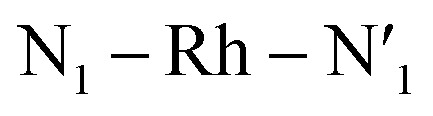
 axis. A second IC process from the higher energy ^3^MC_4_ state towards the lower energy ^3^MC_2_ state subsequently occurs in less than 500 fs (*t*_Ia_ mark), after temporarily reaching a maximum Rh – N bond stretching of 2.92 Å. This particular initial distortion could potentially represent a sweet spot for initiating photochemical reactions.

Triplet crossover dynamics was also simulated at a lower temperature of 298 K as shown in Fig. 4b. This simulation was conducted to check for the presence of alternative deactivation channels, and to further assess the crossover dynamics as a temperature-dependent process. In the 298 K simulation, the ^3^LC lifetime was extended significantly to ∼17 ps in order to successfully capture a crossover event.

While the limited simulation data precludes a reliable Arrhenius analysis that can be correlated quantitatively with the experimentally observed temperature-dependence, it is already notable that the simulations over a range of moderate temperatures shows a clear temperature-dependence of the crossover rate. Furthermore, the transition times in the 1–20 ps time range are consistent with the crossover being an activated process with a small effective transition barrier in basic agreement with a relaxed minimum energy path (details in ESI†). Similar to the 330 K simulation, however, it can be noted that the actual crossover transition remains fast as observed in the rapid accumulation of spin density on the metal center at 17 440 fs. Subsequent geometry relaxation is once again observed after the transition, confirmed by the Rh – N_1_ bond stretching to 2.6 Å. Although the ^α^SOMO orbital 2b shows population of the ^3^MC_3_ state rather than the ^3^MC_4_ observed in the previous simulated temperature, the ^3^LC excited state is still deactivated through a higher energy MC state. This initial population of the ^3^MC_3_ state is rapidly converted into the ^3^MC_4_ excited state in ∼900 fs (as supported by 3b^α^SOMO orbital). An observed second populated ^3^MC_4_ state achieves even longer lifetimes up to ∼2300 fs (*t*_Ib_ mark), before finally deactivating to the calculated lower energy ^3^MC_2_ excited state (*t*_IIb_ mark).

**Fig. 5:**
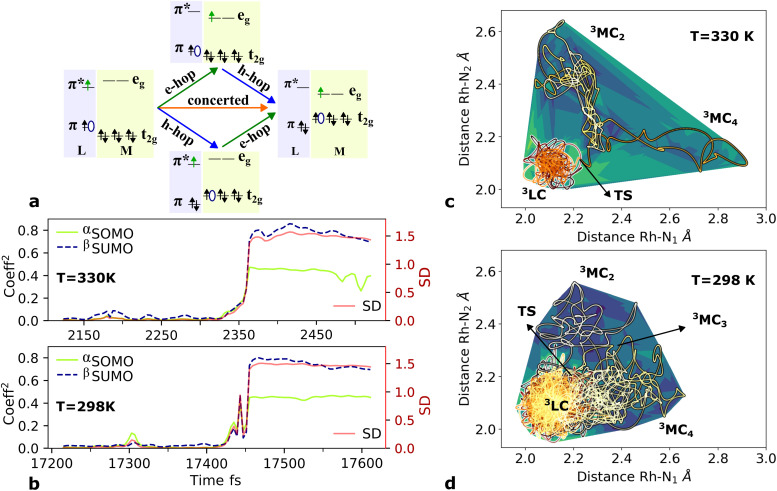
(a) ^3^LC → ^3^MC crossover mechanism representation for the concerted (orange arrow) and two step-wise pathways. Electron hopping (e-hop) is represented with a green arrow and hole hopping (h-hop) is represented with a blue arrow. (b) Normalized square coefficients for the metal d-atomic-orbitals in the ^α^SOMO (green) and ^β^SUMO (blue) during a time frame of 400 fs together with the metal spin density (red) at 330 K and 298 K. AIMD trajectories representation of Rh – N_1_ and Rh – N_2_ bonds at (c) 330 K, (d) 298 K. The contour represents the energy relative to the lowest energy point, and the trajectory color represents the simulation time. The various identified ^3^MC states as well as the crossover steps are indicated by arrows.

Next, we consider the electronic rearrangement taking place during the crossover ^3^LC → ^3^MC in greater detail. This transition is clearly shown by the simulations to proceed as a two-electron process involving the two semi-populated frontier orbitals, similar to what can be expected from a basic intramolecular excitation energy transfer perspective. In particular, the transition involves one electron hopping from the higher energy π* on the initially excited phen ligand to one of the metal centered e_g_ orbitals, together with a hole hopping from the lower-lying occupied π level on the same phen ligand to one of the t_2g_ orbitals on the metal (π → d). The metal center activation can in principle be achieved in a concerted or step-wise fashion *via* electron hopping followed by hole hopping or the equivalent reverse path as illustrated in Fig. 5a. In order to resolve the ^3^LC → ^3^MC mechanism in detail from the simulations, the weights of the molecular orbital coefficients from the metal d-atomic-orbitals in the ^α^SOMO and beta semi-unoccupied molecular orbital (^β^SUMO) were tracked for a series of snapshots with an interval of 4 fs during a 400 fs time window around the transition region (shown in Fig. 5b for 330 K and 298 K and a movie representation for the 298 K simulation in the ESI†). Minor metal orbital contributions to both ^α^SOMO and ^β^SUMO for the initial part of the simulation agrees with a ^3^LC nature during the early part of the reaction window, but quickly ascends up to ∼0.4 for the ^α^SOMO and to ∼0.7 in the ^β^SUMO as the transition progresses. An appreciable increase in atomic d-orbital contributions in the relevant frontier molecular orbitals is observed simultaneously for the ^α^SOMO and ^β^SUMO, and also matches closely with the reported increase of spin density on the metal. These findings support that the two-electron transition indeed proceeds in a concerted fashion without any significant involvement of transiently populated one-electron charge-transfer states. While the two-electron transfer is in good accordance with the net resulting experimental observation of the crossover activation of the ^3^MC state, it is notable that the structural distortions coupled with intramolecular coulombic and exchange interactions promotes the simultaneous transition of both the electron and the hole all the way down to ultrafast timescales, rather than the crossover being driven by one or the other electronic transitions.

Finally, the AIMD simulations also highlight the importance of statistical factors influencing the crossover dynamics beyond what can be extracted from static calculations of transition barriers and scans of reaction paths. In particular, the simulations indicate a clear significance of entropy factors related to the accessible PES volume for the different states. This effective area, as an intrinsic characteristic of the distinct excited states dynamics for both sampled temperatures, are already revealed in the trajectories within the phen M–L bonds during the simulation time as represented in Fig. 5c and d. The population area of the ^3^LC state at 330 K is significantly more narrowly localized (in terms of phen M–L bond distortions) than at 298 K so that the transition state (TS) is essentially reached without any initial geometry stretching. In contrast, a TS at 298 K is only reached by bond stretching after significantly longer state sampling. The two highest energy ^3^MC excited states behave as important intermediate states, reflecting the complexity of multi-step deactivation paths towards the ultimate population of the lowest ^3^MC_2_ state.

The larger accessible volume in the ^3^MC product state corresponds an increase of the reaction entropy. Since the ^3^LC → ^3^MC transition is mainly ruled by M–L Jahn–Teller distortions, we assess the change in accessible coordinate space in the simulations by the six M–L bond distributions. In this approximation, the accessible volume of each state can be expressed as the product of the standard deviations of each M–L bond (*δl*_*i*_) by1
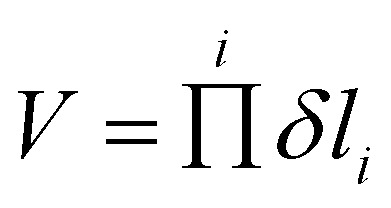


The entropy change of the crossover reaction (Δ*S*_r_) then relates with the Boltzmann constant *k*_B_ and the partition function in terms of volume changes between the ^3^MC state (*V*_MC_) and the ^3^LC state (*V*_LC_) as2
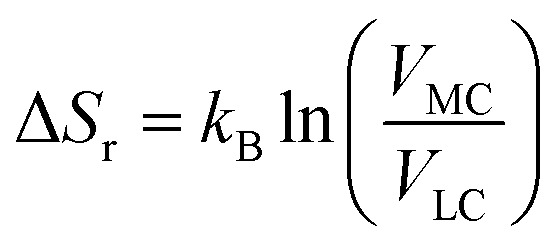


The standard deviations for the six M–L bonds for the studied system [Rh(iii)(phen)_2_(NH_3_)_2_]^3+^ complex together with the final active volume *V* (calculated using eqn (1)) in the three sampled temperatures (298 K, 200 K and 170 K) are tabulated in Table SI.4.† For the 298 K simulations, where equilibration between different local ^3^MC minima takes place, the reaction entropy for a ^3^LC → ^3^MC process, Δ*S*_r_, is estimated to be 41.86 J K^−1^ mol^−1^. The Δ*S*_r_ calculated from AIMD trajectories agrees well with the corresponding energies calculated from static quantum chemical calculations ∼40 J K^−1^ mol^−1^ (Table SI.5†), validating the employed protocol and emphasizing that the entropy change is mainly driven by the M–L bond distortions. This provides a considerable entropy contribution to the reaction free energy for the finely balanced ^3^LC–^3^MC dynamics. This also highlights that entropy factors should be considered to properly assess the driving force and reversibility for these activated crossover processes beyond what has traditionally been done to assess the temperature-dependent activation behaviour in these complexes.^19^

## 4 Conclusions

AIMD simulations were used to investigate ^3^LC → ^3^MC crossover dynamics in the rhodium complex [Rh(iii)(phen)_2_(NH_3_)_2_]^3+^ on picosecond timescales. Initial results from quantum chemical calculations revealed a clear difference in the triplet potential energy landscape between well-defined ^3^LC states on the phen ligands on one hand, and a more complex ^3^MC potential energy landscape comprising several local minima with distinct structural and electronic characteristics on the other hand.

The AIMD simulations provide further insight into the multidimensional dynamics taking place on the complex triplet PES. First, ^3^LC internal state dynamics reveal a narrow and well-defined range of accessible geometries closely constrained near the ground state geometry. In contrast, ^3^MC dynamics is revealed to involve significant structural mobility on a flat ^3^MC PES that includes facile transitions between several Jahn–Teller distorted minima. The MC dynamics notably includes fast transitions through higher-energy MC states connecting different low-energy parts of the full ^3^MC PES. Notably, the large difference in thermally accessible coordinate space between the LC and MC parts of the triplet PES indicates that entropic effects will have a significant influence on the free energy balance and effective driving force governing the ^3^LC → ^3^MC crossover dynamics.

Simulations of the ^3^LC → ^3^MC process at different temperatures showed a significant influence of the temperature on the crossover rate, consistent with an activated process characterized by a small energy barrier. The simulations also reveal further details of the triplet dynamics proceeding *via* a complex cascade involving several local ^3^MC states following the initial crossover from the ^3^LC state. Analysis of the AIMD simulations also enabled an in-depth assessment of the electric rearrangements taking place during the ^3^LC → ^3^MC crossover. In particular, the ^3^LC → ^3^MC transition is seen to involve a rapid redistribution of both the excited electron and the accompanying hole from one of the phen ligands to the metal center that takes place in a fully concerted fashion on a sub-picosecond timescale. This corresponds to a spin-allowed intramolecular two-electron excitation energy transfer process that takes place dynamically as a structure-driven weakening of the ligand field splitting of the metal center. It is interesting to consider as a topic for further investigation that the two-electron nature of this process appears to provide a fundamentally different set of opportunities and limitations for tuning of the excited state landscape compared to the corresponding one-electron case encountered in more widely studied ^3^MLCT → ^3^MC deactivation processes.

Finally, the in-depth understanding of this ^3^LC → ^3^MC transition provides useful insights for further developments of transition metal complexes that aim to utilize ^3^MC states for photochemical applications more broadly. In particular, ^3^LC → ^3^MC crossover is confirmed to provide a promising approach to overcome problems associated with weak absorption and ultrafast initial energy losses from direct excitations into ^3^MC states more generally, as long as the full complexity of the triplet potential energy landscape and associated dynamics revealed by simulations is taken into consideration.

## Data availability

DFT energies and geometry details, trajectories and electron structure analysis of the MD simulations, NEB calculation analysis, and cartesian coordinates of the optimized structures are available in the ESI.† A ^α^SOMO ^3^LC → ^3^MC transition dynamics MP4 movie at 298 K is also available in the ESI.†

## Author contributions

PP supervised the project and IBL performed the calculations. PP and IBL contributed to the manuscript preparation.

## Conflicts of interest

There are no conflicts to declare.

## Supplementary Material

SC-014-D3SC04381A-s001

SC-014-D3SC04381A-s002

## References

[cit1] Sinha N., Wenger O. S. (2023). J. Am. Chem. Soc..

[cit2] Förster C., Heinze K. (2020). Chem. Soc. Rev..

[cit3] Zigmantas D., Polívka T., Persson P., Sundström V. (2022). Chem. Phys. Rev..

[cit4] Prier C. K., Rankic D. A., MacMillan D. W. C. (2013). Chem. Rev..

[cit5] Wagenknecht P. S., Ford P. C. (2011). Coord. Chem. Rev..

[cit6] Ting S. I., Garakyaraghi S., Taliaferro C. M., Shields B. J., Scholes G. D., Castellano F. N., Doyle A. G. (2020). J. Am. Chem. Soc..

[cit7] Förster C., Heinze K. (2022). Chem. Phys. Rev..

[cit8] Welin E. R., Le C., Arias-Rotondo D. M., McCusker J. K., MacMillan D. W. C. (2017). Science.

[cit9] Otto S., Grabolle M., Förster C., Kreitner C., Resch-Genger U., Heinze K. (2015). Angew. Chem., Int. Ed..

[cit10] Woodhouse M. D., McCusker J. K. (2020). J. Am. Chem. Soc..

[cit11] Kaufhold S., Rosemann N. W., Chábera P., Lindh L., Bolaño Losada I., Uhlig J., Pascher T., Strand D., Wärnmark K., Yartsev A., Persson P. (2021). J. Am. Chem. Soc..

[cit12] Sinha N., Pfund B., Wegeberg C., Prescimone A., Wenger O. S. (2022). J. Am. Chem. Soc..

[cit13] Chan A. Y., Ghosh A., Yarranton J. T., Twilton J., Jin J., Arias-Rotondo D. M., Sakai H. A., McCusker J. K., MacMillan D. W. C. (2023). Science.

[cit14] Alowakennu M. M., Ghosh A., McCusker J. K. (2023). J. Am. Chem. Soc..

[cit15] Eastham K., Scattergood P. A., Chu D., Boota R. Z., Soupart A., Alary F., Dixon I. M., Rice C. R., Hardman S. J. O., Elliott P. I. P. (2022). Inorg. Chem..

[cit16] Soupart A., Alary F., Heully J. L., Elliott P. I., Dixon I. M. (2020). Coord. Chem. Rev..

[cit17] Thomas T., Crosby G. (1971). J. Mol. Spectrosc..

[cit18] Petersen J. D., Watts R. J., Ford P. C. (1976). J. Am. Chem. Soc..

[cit19] Indelli M. T., Scandola F. (1990). Inorg. Chem..

[cit20] Thomas T., Watts R., Crosby G. (1973). J. Chem. Phys..

[cit21] Brozik J., Crosby G. (1996). Chem. Phys. Lett..

[cit22] Carstens D., Crosby G. (1970). J. Mol. Spectrosc..

[cit23] Miki H., Shimada M., Azumi T., Brozik J., Crosby G. (1993). J. Phys. Chem..

[cit24] Indelli M. T., Carioli A., Scandola F. (1984). J. Phys. Chem..

[cit25] Indelli M. T. , Chiorboli C. and Scandola F., Photochemistry and Photophysics of Coordination Compounds I, 2007, pp. 215–255

[cit26] Barigelletti F., Sandrini D., Maestri M., Balzani V., Von Zelewsky A., Chassot L., Jolliet P., Maeder U. (1988). Inorg. Chem..

[cit27] Sousa C., Llunell M., Domingo A., de Graaf C. (2018). Phys. Chem. Chem. Phys..

[cit28] Iuchi S., Koga N. (2016). Phys. Chem. Chem. Phys..

[cit29] Pápai M. (2021). Inorg. Chem..

[cit30] Daku L. M. L. (2019). Phys. Chem. Chem. Phys..

[cit31] Zederkof D. B., Møller K. B., Nielsen M. M., Haldrup K., González L., Mai S. (2022). J. Am. Chem. Soc..

[cit32] Mai S., González L. (2019). Chem. Sci..

[cit33] Šrut A., Mai S., Sazanovich I. V., Heyda J., Vlček A., González L., Záliš S. (2022). Phys. Chem. Chem. Phys..

[cit34] Heindl M., Hongyan J., Hua S. A., Oelschlegel M., Meyer F., Schwarzer D., González L. (2021). Inorg. Chem..

[cit35] Freitag L., Gonzalez L. (2014). Inorg. Chem..

[cit36] Hsu D. J., Leshchev D., Kosheleva I., Kohlstedt K. L., Chen L. X. (2020). J. Chem. Phys..

[cit37] Bolletta F., Rossi A., Barigelletti F., Dellonte S., Balzani V. (1981). Gazz. Chim. Ital..

[cit38] Nishizawa M., Suzuki T., Sprouse S., Watts R., Ford P. (1984). Inorg. Chem..

[cit39] Becke A. D. (1993). J. Chem. Phys..

[cit40] Reiher M., Salomon O., Hess B. A. (2001). Theor. Chem. Acc..

[cit41] McLean A. D., Chandler G. S. (1980). J. Chem. Phys..

[cit42] Andrae D., Haeussermann U., Dolg M., Stoll H., Preuss H. (1990). Theor. Chim. Acta.

[cit43] Scalmani G., Frisch M. J. (2010). J. Chem. Phys..

[cit44] Frisch M. J. , Trucks G. W., Schlegel H. B., Scuseria G. E., Robb M. A., Cheeseman J. R., Scalmani G., Barone V., Petersson G. A., Nakatsuji H., Caricato M., Li X., Hratchian H. P., Izmaylov A. F., Bloino J., Zheng G., Sonnenberg J. L., Hada M., Ehara M., Toyota K., Fukuda R., Hasegawa J., Ishida M., Nakajima T., Honda Y., Kitao O., Nakai H., Vreven T., Montgomery Jr J. A., Peralta J. E., Ogliaro F., Bearpark M. J., Heyd J. J., Brothers E. N., Kudin K. N., Staroverov V. N., Keith T., Kobayashi R., Normand J., Raghavachari K., Rendell A. P., Burant J. C., Iyengar S. S., Tomasi J., Cossi M., Rega N., Millam J. M., M. K., Knox J. E., Cross J. B., Bakken V., Adamo C., Jaramillo J., Gomperts R., Stratmann R. E., Yazyev O., Austin A. J., Cammi R., Pomelli C., Ochterski J. W., Martin R. L., Morokuma K., Zakrzewski V. G., Voth G. A., Salvador P., Dannenberg J. J., Dapprich S., Daniels A. D., Farkas O., Foresman J. B., Ortiz J. V., Cioslowski J. and Fox D. J., Gaussian 09 Revision A.02, Gaussian Inc., Wallingford CT, 2009

[cit45] Schlegel H. B., Iyengar S. S., Li X., Millam J. M., Voth G. A., Scuseria G. E., Frisch M. J. (2002). J. Chem. Phys..

[cit46] Tsai C. N., Mazumder S., Zhang X. Z., Schlegel H. B., Chen Y. J., Endicott J. F. (2015). Inorg. Chem..

[cit47] Dixon I. M., Heully J. L., Alary F., Elliott P. I. (2017). Phys. Chem. Chem. Phys..

[cit48] Borg O. A., Godinho S. S. M. C., Lundqvist M. J., Lunell S., Persson P. (2008). J. Phys. Chem. A.

