# Supplementary material for: Ligand-centered to metal-centered activation of a Rh(iii) photosensitizer revealed by ab initio molecular dynamics simulations
Source: Chem Sci. 2023 Nov 2;14(47):13713–21. doi: 10.1039/d3sc04381a (PMC10699561; doi:10.1039/d3sc04381a)
Supplement: SC-014-D3SC04381A-s001 [file SC-014-D3SC04381A-s001.pdf]

Electronic Supplementary Material (ESI) for Chemical Science.
This journal is © The Royal Society of Chemistry 2023

Supplemental Information for "Ligand-Centered to Metal-Centered Activation of a Rh(III) Photosensitizer revealed by Ab Initio Molecular Dynamics Simulations"

Iria Bolaño Losada[a] and Petter Persson*[a]
Division of Computational Chemistry, Department of Chemistry, Lund University, Box 124, SE-22100 Lund, Sweden.

List of Figures

SI.1 Energy and average metal-ligand bond distances of ^{3}LC and ^{3}MC states 2
SI.2 ^{3}LC and ^{3}MC states $^{\alpha}$SOMO orbital 3
SI.3 ^{3}LC metal-ligand bond distance distributions 4
SI.4 ^{3}LC dipole moment histogram 4
SI.5 ^{3}LC atom spin density progression 5
SI.6 Root mean square deviations 6
SI.7 ^{3}MC state Phenanthroline bite angles 7
SI.8 AIMD trajectories at 170 K 8
SI.9 AIMD trajectories at 200 K 8
SI.10 ^{3}LC to ^{3}MC minimum energy path 9

List of Tables

SI.1 ^{3}LC emission energy 2
SI.2 Metal-ligand bond distances 3
SI.3 ^{3}LC and ^{3}MC states energies 3
SI.4 Bonds standard deviations and effective volumes 8
SI.5 ^{3}LC to ^{3}MC crossover entropy change 9
SI.6 ^{3}LC coordinates at B3LYP*/3-21G/PCM(Acetonitrile) 10
SI.7 $^3MC_1$ coordinates at B3LYP*/3-21G/PCM(Acetonitrile) 11
SI.8 $^3MC_2$ coordinates at B3LYP*/3-21G/PCM(Acetonitrile) 12
SI.9 $^3MC_3$ coordinates at B3LYP*/3-21G/PCM(Acetonitrile) 13
SI.10 ^{3}LC coordinates at B3LYP*/6-311G(d,f)/PCM(Acetonitrile) 14
SI.11 $^3MC_1$ coordinates at B3LYP*/6-311G(d,f)/PCM(Acetonitrile) 15
SI.12 $^3MC_2$ coordinates at B3LYP*/6-311G(d,f)/PCM(Acetonitrile) 16
SI.13 $^3MC_3$ coordinates at B3LYP*/6-311G(d,f)/PCM(Acetonitrile) 17
SI.14 $^3MC_4$ coordinates at B3LYP*/6-311G(d,f)/PCM(Acetonitrile) 18
SI.15 ^{3}LC frequency list (cm^{-1}) at B3LYP*/3-21G/PCM(Acetonitrile) 19
SI.16 $^3MC_1$ frequency list (cm^{-1}) at B3LYP*/3-21G/PCM(Acetonitrile) 20
SI.17 $^3MC_2$ frequency list (cm^{-1}) at B3LYP*/3-21G/PCM(Acetonitrile) 21
SI.18 $^3MC_3$ frequency list (cm^{-1}) at B3LYP*/3-21G/PCM(Acetonitrile) 22
SI.19 ^{3}LC frequency list (cm^{-1}) at B3LYP*/6-311G(d,f)/PCM(Acetonitrile) 23
SI.20 $^3MC_1$ frequency list (cm^{-1}) at B3LYP*/6-311G(d,f)/PCM(Acetonitrile) 24
SI.21 $^3MC_2$ frequency list (cm^{-1}) at B3LYP*/6-311G(d,f)/PCM(Acetonitrile) 25
SI.22 $^3MC_3$ frequency list (cm^{-1}) at B3LYP*/6-311G(d,f)/PCM(Acetonitrile) 26
SI.23 $^3MC_4$ frequency list (cm^{-1}) at B3LYP*/6-311G(d,f)/PCM(Acetonitrile) 27

I. COMPUTATIONAL DETAILS

The Ab initio molecular dynamics (AIMD) calculations were carried out using an UltraFine integration grid, and the nature of the different optimized triplet excited states were confirmed by evaluating the spin density distribution. Full SCF convergence (FullSCF keyword) and allowing quadratic convergence (XQC) if needed was requested for each MD step allowing the equations of motions to be updated with a typical step size of 0.5 fs, and the fictitious electronic mass of 0.1 amu. In some AIMD simulations, the step size was reduced to 0.4 fs for validation purposes, or to avoid nonphysical bond displacement. All systems were initially equilibrated for 2.5 ps in the case of the ^{3}LC state, and 3.5 ps for the ^{3}MC states as suggested from analyzing the root mean squared deviation (RMSD) stability over time. Subsequent MD production trajectories were recorded and analyzed during a total time of 15 ps for a full analysis at 200 K, as well as shorter simulations of 7.5 ps for the temperature study at 170 K and 298 K. A thermostat was set to control the temperature (IOp(1/8x) options) allowing deviations of maximum 50 K, reevaluating and rescaling every 10 steps all along the simulation. An AIMD simulation at 330 K for studying the crossover process was initially set at the ^{3}LC state and equilibrated during 2.5 ps, followed by production trajectories and sampling collected until ^{3}LC $\rightarrow$ ^{3}MC conversion had been clearly established. We have performed minimum energy path calculations between the ^{3}LC and ^{3}MC$_2$ states structures by linearly interpolating 20 intermediate points, with average steps of the six metal-ligand bonds of approximately 0.004 Å , and subsequently relaxing the structure with constrained metal-ligand bonds, at the same B3LYP*/3-21G/PCM(Water) level of theory.

TABLE SI.1: ^{3}LC emission energy (in eV) calculated with 6-311G(d,f) and 3-21G basis sets for a series of functionals. Experimental energy (Exp) is also tabulated.

	^{3}LC Emission (eV)	
State	6-311G(d,f)	3-21G
B3LYP	2.382	2.446
B3LYP*	2.402	2.443
O3LYP	2.415	2.475
PBE	2.344	2.424
PBE0	2.357	2.434
TPSSh	2.323	3.386
M06	2.350	2.337
BMK	2.493	2.651
Exp	2.38	2.38

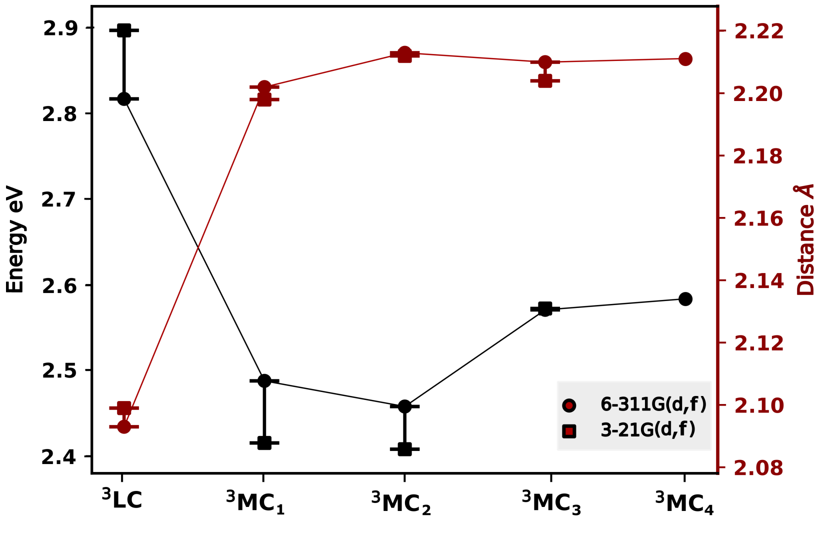

FIG. SI.1: Average metal-ligand bond distances in Å (red) and energies respect to to the ground state in eV (black) for the hexa-coordinated complex [Rh(III)(phen)$_2$(NH$_3$)$_2$]$^{3+}$ for ^{3}LC, ^{3}MC$_1$, ^{3}MC$_2$, ^{3}MC$_3$ and ^{3}MC$_4$ states. (Dot markers) B3LYP*/6-311G(d)/PCM(water), (Square markers) B3LYP*/3-21G(d)/PCM(water).

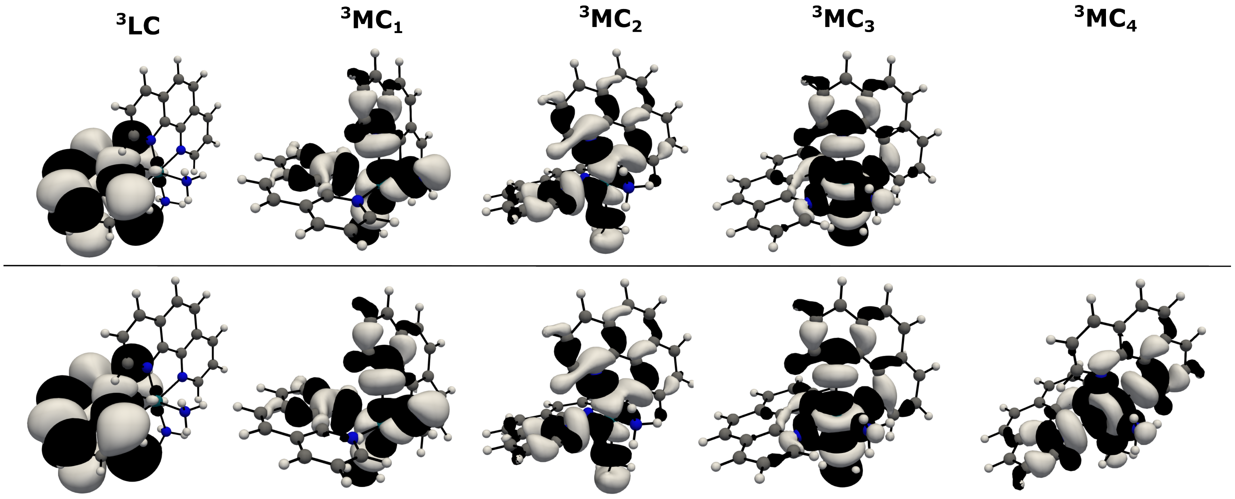

FIG. SI.2: $^{\alpha}$SOMO orbital representation of the ^{3}LC, ^{3}MC$_1$, ^{3}MC$_2$, ^{3}MC$_3$ and ^{3}MC$_4$ excited states (top panel) B3LYP*/3-21G(d)/PCM(water), (lower panel) B3LYP*/6-311G(d)/PCM(water).

TABLE SI.2: Metal-ligand bond distances Rh-N$_1$, Rh-N'$_1$, Rh-N$_2$, Rh-N'$_2$, Rh-N$_a$, Rh-N'$_a$ as well as average distances in Å in the optimized hexa-coordinated $[Rh(III)(phen)_2(NH_3)_2]^{3+}$complex in the ^{3}LC and ^{3}MC states at B3LYP*/6-311G(d,f)/PCM(water) and B3LYP*/3-21G(d,f)/PCM(water) levels of calculation.

	^3LC		$^3MC_1$		$^3MC_2$		$^3MC_3$		$^3MC_4$	
Bond	6-311G(d,f)	3-21G	6-311G(d,f)	3-21G	6-311G(d,f)	3-21G	6-311G(d,f)	3-21G	6-311G(d,f)	3-21G
Rh-N1	2.084	2.089	2.088	2.089	2.198	2.193	2.185	2.168	2.413	N/A
Rh-N1'	2.090	2.095	2.088	2.088	2.277	2.268	2.094	2.102	2.399	N/A
Rh-N2	2.078	2.083	2.290	2.327	2.376	2.413	2.332	2.309	2.088	N/A
Rh-N2'	2.079	2.085	2.290	2.327	2.091	2.088	2.116	2.147	2.084	N/A
Rh-Na	2.114	2.122	2.216	2.191	2.205	2.207	2.403	2.350	2.141	N/A
Rh-Na'	2.112	2.122	2.217	2.191	2.118	2.112	2.129	2.148	2.139	N/A
Average	2.093	2.099	2.202	2.198	2.213	2.212	2.210	2.204	2.211	N/A

TABLE SI.3: Relative energies (E, in eV) of the ^{3}LC and ^{3}MC states with respect to the ^{1}GS of the $[Rh(III)(phen)_2(NH_3)_2]^{3+}$complex at the B3LYP*/6-311G(d,f)/PCM(water) and B3LYP*/3-21G(d,f)/PCM(water) levels of calculation. Gibbs free energies (ΔG, in eV and kJ/mol) with respect to the ^{1}GS, dipole moment (Debye) and the frequency (cm^{-1}) of lower energy are also included for each state.

	E (eV)		ΔG (eV)/(kJ/mol)		μ (D)		ρ_M		f (cm^{-1})	
State	6-311G(d,f)	3-21G	6-311G(d,f)	3-21G	6-311G(d,f)	3-21G	6-311G(d,f)	3-21G	6-311G(d,f)	3-21G
^{3}LC	2.817	2.897	2.615/252.309	2.689/259.449	7.568	7.862	0.004	0.003	26.3764	26.5870
^{3}MC$_1$	2.488	2.415	2.232/215.355	2.202/212.461	7.732	8.084	1.448	1.443	25.706	26.483
^{3}MC$_2$	2.458	2.408	2.277/219.697	2.233/215.452	10.682	10.743	1.484	1.475	22.808	30.922
^{3}MC$_3$	2.571	2.572	2.283/220.276	2.272/219.215	7.828	8.448	1.488	1.478	22.401	13.908
^{3}MC$_4$	2.583	N/A	2.347/226.451	N/A	7.652	N/A	1.469	N/A	23.626	N/A

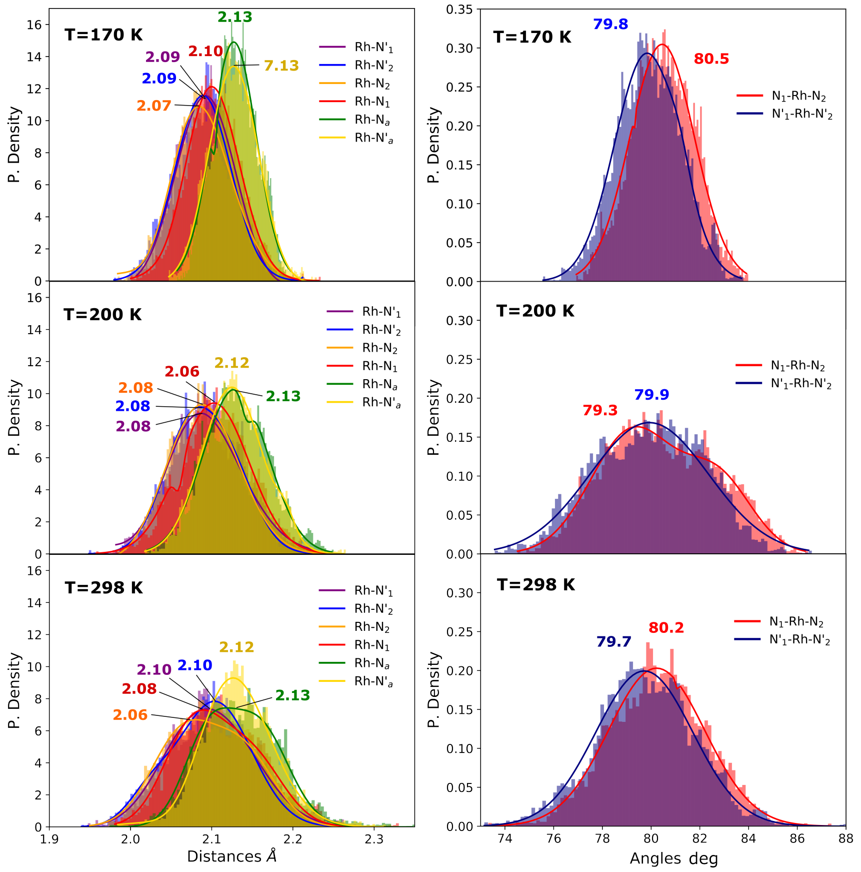

FIG. SI.3: ^{3}LC metal-ligand bond distance distributions in Å for the hexa-coordinated $[Rh(III)(phen)_2(NH_3)_2]^{3+}$ (Rh-N'$_1$ in purple and Rh-N'$_2$ in blue, Rh-N$_1$ in red and Rh-N$_2$ in orange and Rh-N'$_a$ in yellow and Rh-N$_a$ in green) together with phenantroline bite angle distributions α (phen ligand) and β (phen' ligand) in degrees (deg) during 7.5 ps AIMD sampling at (top panel) 170 K, (middle panel), 200 K (bottom panel) 298 K.

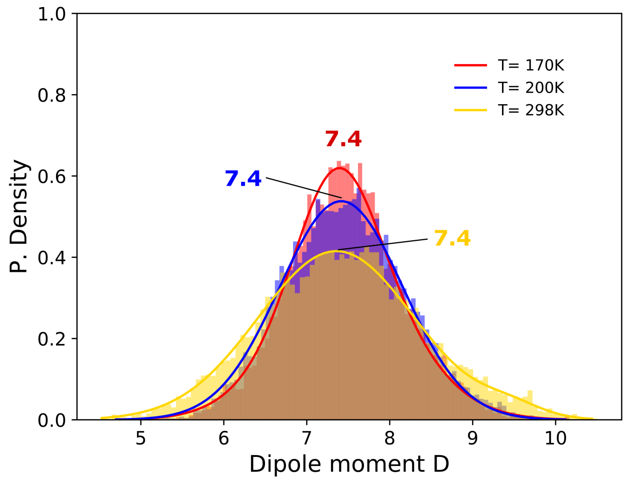

FIG. SI.4: Dipole moment histogram in Debye (D) for the ^{3}LC states in the $[Rh(III)(phen)_2(NH_3)_2]^{3+}$ complex for 7.5 ps of AIMD sampling at (red) 170 K, (blue) 200 K, (yellow) 298 K.

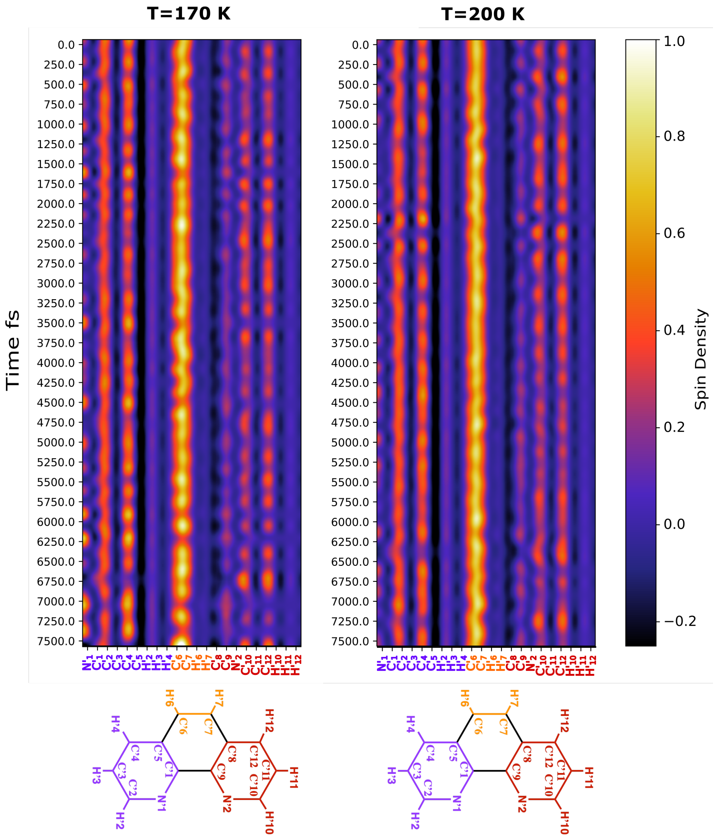

FIG. SI.5: Atom spin density population progression along time in an AIMD simulation of 7500 fs in the phenantroline phen' ligand of the $[Rh(III)(phen)_2(NH_3)_2]^{3+}$ complex at (left) 170 K, (right) 200 K. Atom numbering presented below and color code.

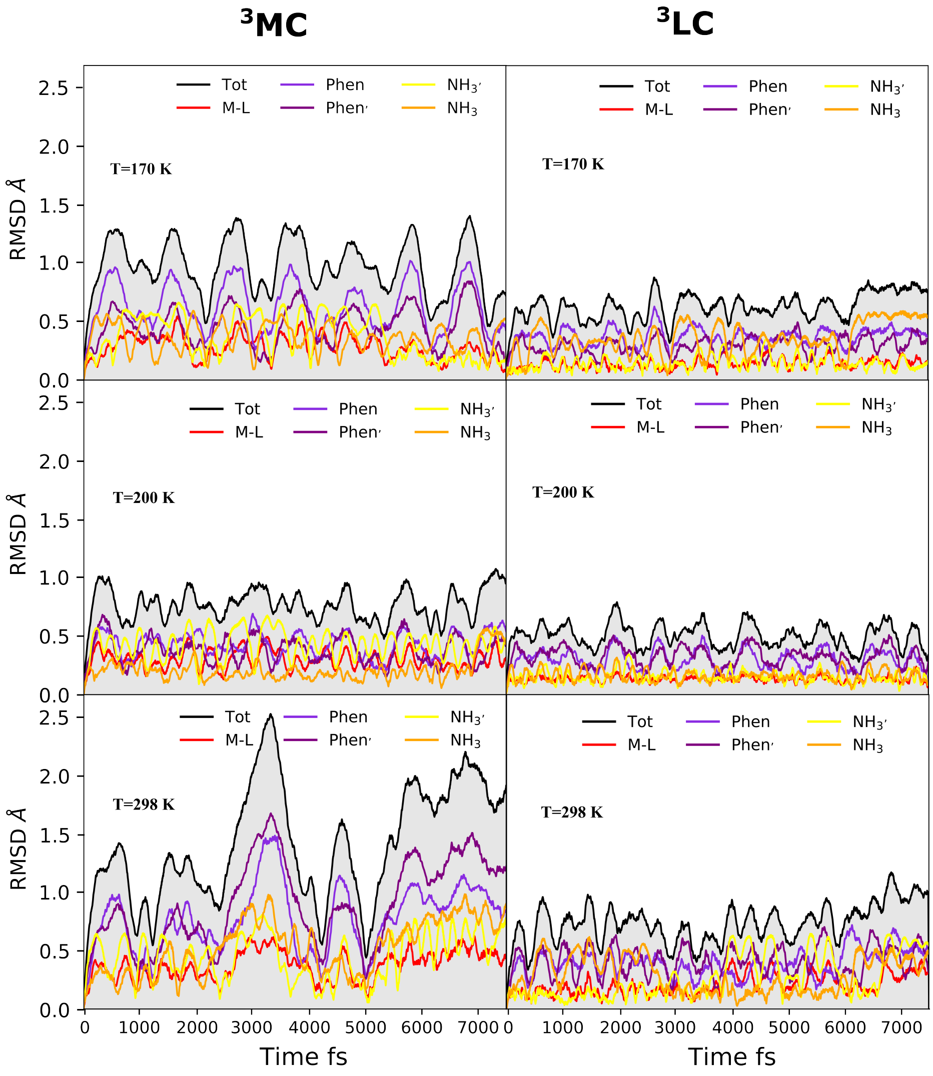

FIG. SI.6: Root mean square deviation (RMSD) plots over time with the corresponding ligand contribution to the total RMSD for the ^{3}MC and ^{3}LC AIMD sampling in the $[Rh(III)(phen)_2(NH_3)_2]^{3+}$ complex at (top panel) 170 K, (middle panel) 200 K, (bottom panel) 298 K

.

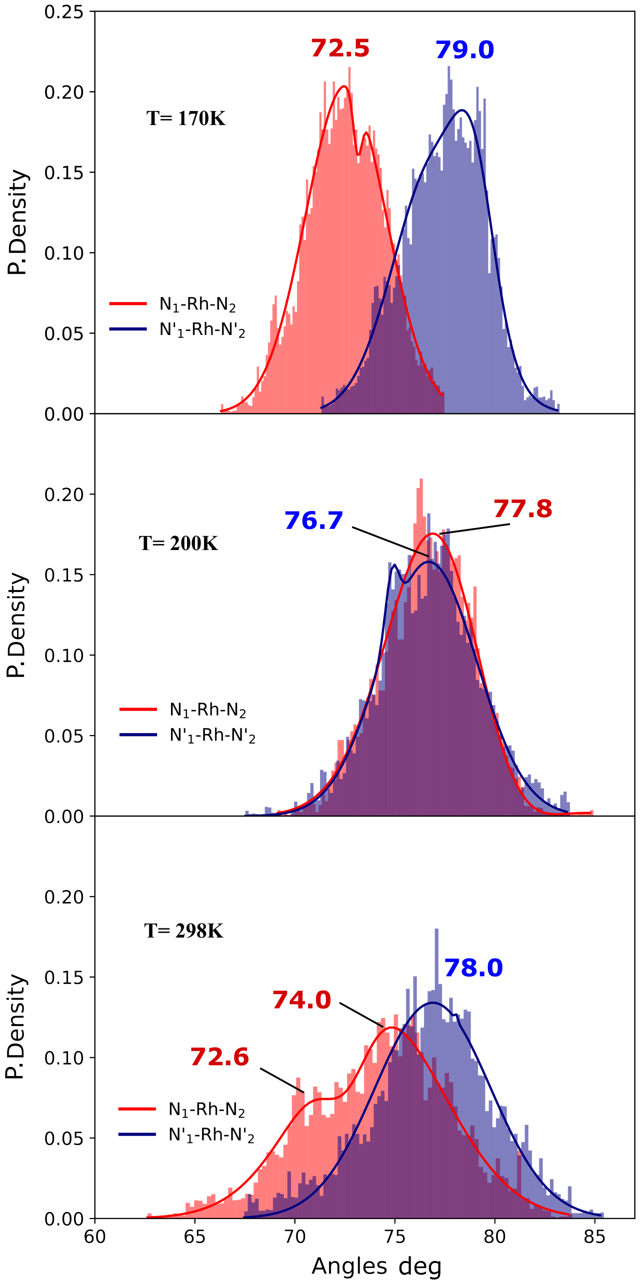

FIG. SI.7: Phenantroline bite angles distributions in degrees (deg) for phen ligand α and phen' ligand β during 7.5 ps AIMD sampling in the ^{3}MC state in the $[Rh(III)(phen)_2(NH_3)_2]^{3+}$ complex at (top panel) 170 K, (middle panel) 200 K, (bottom panel) 298 K.

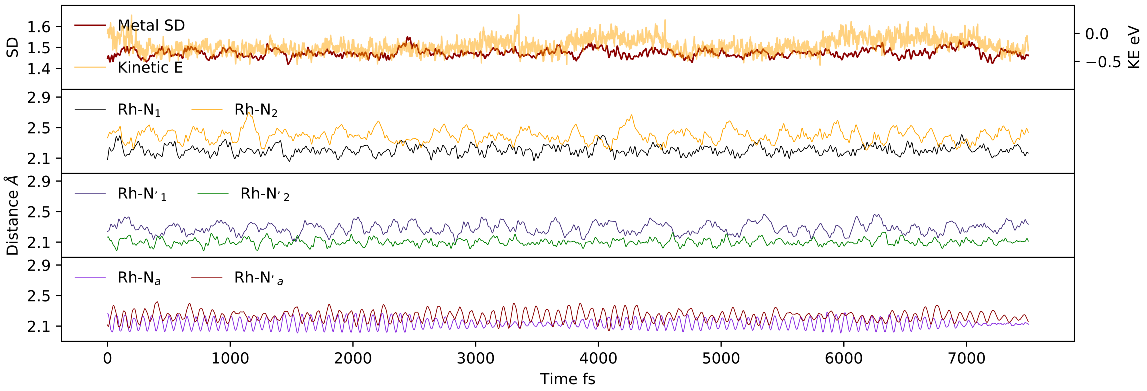

FIG. SI.8: AIMD trajectories over simulation time (7500 fs) at 170 K, (top panel) metal spin density (SD) in red color, (top panel) kinetic energy (KE) fluctuation respect to the first production snapshot in yellow and (lower panels) metal to ligand bond distances (in Å) for both bidentate ligands, phen (Rh-N_1 in black and Rh-N_2 in yellow) and phen' (Rh-N'$_1$ in blue and Rh-N'$_2$ in green) and both amine groups (Rh-N_a in purple and Rh-N'$_a$ in red).

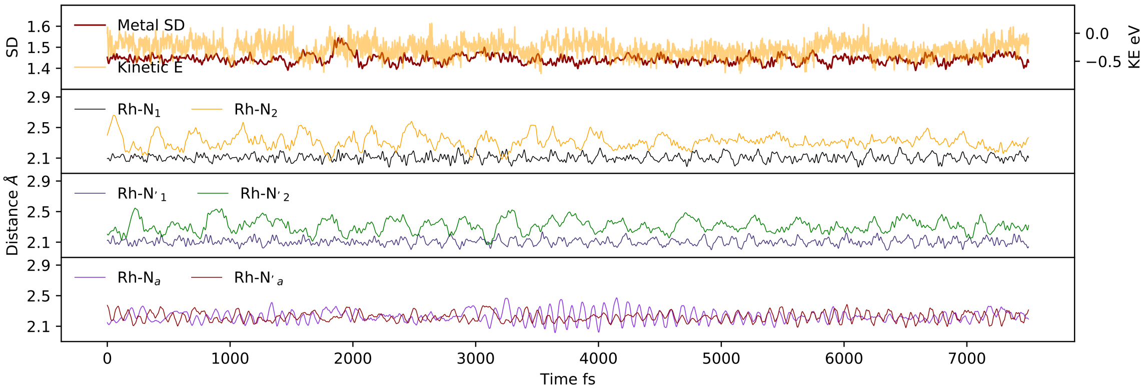

FIG. SI.9: AIMD trajectories over simulation time (7500 fs) at 200 K, (top panel) metal spin density (SD) in red color, (top panel) kinetic energy (KE) fluctuation respect to the first production snapshot in yellow and (lower panels) metal to ligand bond distances (in Å) for both bidentate ligands, phen (Rh-N_1 in black and Rh-N_2 in yellow) and phen'(Rh-N'$_1$ in blue and Rh-N'$_2$ in green) and both amine groups (Rh-N_a in purple and Rh-N'$_a$ in red).

TABLE SI.4: Metal-ligand bond distance standard deviation and effective volumes calculated from a 7.5 ps AIMD simulation in the ^{3}MC and ^{3}LC states at 298 K, 200 K and 170 K.

	^{3}MC			^{3}LC		
δl (Å)	T=298 (K)	T=200 (K)	T=170 (K)	T=298 (K)	T=200 (K)	T=170 (K)
δl_{Rh-N1}	0.1151	0.0408	0.0583	0.0541	0.0405	0.0331
$\delta l_{Rh-N'1}$	0.1360	0.0406	0.0697	0.0509	0.0426	0.0327
δl_{Rh-N2}	0.1537	0.0821	0.0837	0.0512	0.0407	0.0373
$\delta l_{Rh-N'2}$	0.1248	0.0798	0.0405	0.0510	0.0405	0.0346
δl_{Rh-Na}	0.0904	0.0545	0.0675	0.0442	0.0399	0.0257
$\delta l_{Rh-N'a}$	0.0813	0.0640	0.0619	0.0451	0.0352	0.0289
V (Å^6)	2.21x10^{-6}	3.79x10^{-8}	5.76x10^{-8}	1.44x10^{-8}	4.00x10^{-9}	1.05x10^{-9}

TABLE SI.5: ^{3}LC to ^{3}MC crossover entropy change at AIMD sampling temperatures of 298 K, 200 K and 170 K.

T (K)	ΔS (J/Kmol)
298	41.8659
200	18.6980
170	33.3058

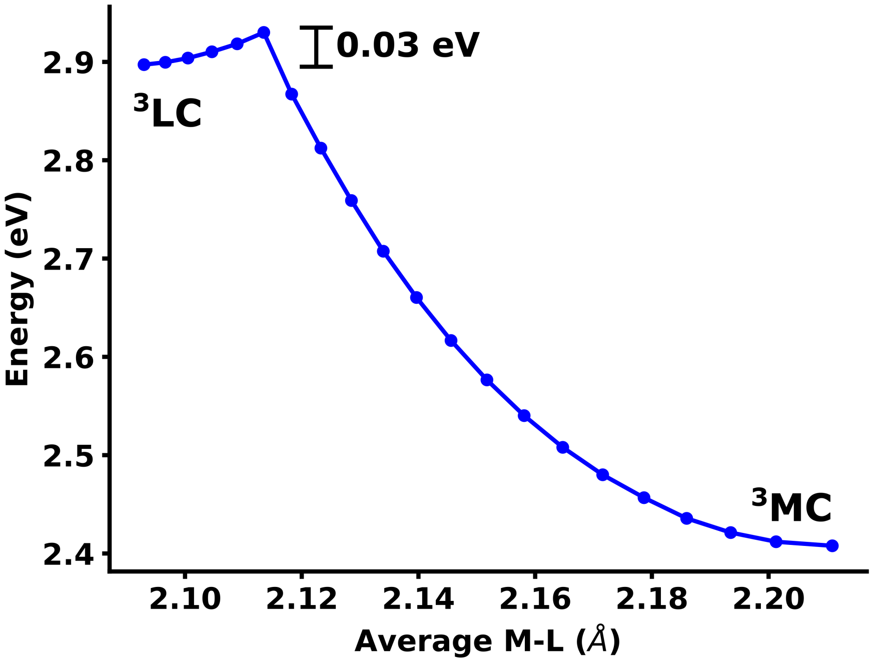

FIG. SI.10: Minimum energy path between the ^{3}LC and the $^3MC_2$ optimized minimum geometries at the B3LYP*/3-21G(d,f)/PCM(water) level of theory. The energies, in eV, are given with respect to the ground state energy.

TABLE SI.6: ^{3}LC coordinates at B3LYP*/3-21G/PCM(Acetonitrile)

53

N 1.929209 0.712291 -0.968539
C 2.697730 0.103442 -0.014147
C 4.045458 0.458517 0.210298
C 4.589175 1.481711 -0.596223
C 3.800510 2.085231 -1.553704
C 2.469087 1.676109 -1.716088
C 2.088405 -0.921869 0.765020
C 2.828215 -1.588484 1.765072
C 4.194837 -1.210878 1.977235
C 4.778456 -0.230186 1.231969
C 2.164777 -2.596884 2.497794
C 0.847430 -2.889854 2.212944
C 0.179962 -2.182979 1.201410
N 0.783822 -1.225258 0.498858
Rh -0.013319 -0.057751 -1.025247
N -0.798923 1.269514 0.366780
C -2.075478 1.019519 0.678325
C -2.808108 1.812427 1.618117
C -2.106302 2.902016 2.222943
C -0.789165 3.134068 1.881328
C -0.147584 2.311027 0.952767
C -2.704488 -0.116237 0.009973
C -4.069509 -0.448406 0.285715
C -4.768935 0.332342 1.200019
C -4.131498 1.477098 1.877517
C -4.611082 -1.574067 -0.412731
C -3.806077 -2.273727 -1.298314
C -2.487964 -1.888483 -1.508191
N -1.950233 -0.807219 -0.847691
N 0.685890 -1.461997 -2.442406
H -0.852841 -2.399110 0.958576
H 0.309310 -3.659452 2.754407
H 4.759188 -1.726220 2.748259
H 2.696327 -3.134397 3.277310
H 5.622313 1.785307 -0.457562
H 5.814576 0.047040 1.400474
H 1.837940 2.142868 -2.462004
H 4.185412 2.873917 -2.189810
H 0.881017 2.471477 0.662313
H -0.232618 3.952952 2.323536
H -4.713809 2.055730 2.585879
H -2.616569 3.534205 2.942351
H -5.641478 -1.867288 -0.241926
H -5.804829 0.105647 1.427270
H -1.840704 -2.422601 -2.188576
H -4.189807 -3.131686 -1.839729
H 0.070107 -1.583629 -3.246225
H 1.592386 -1.188539 -2.822941
H 0.819975 -2.385043 -2.028640
N -0.727783 1.180795 -2.580392
H -0.870050 2.141368 -2.267386
H -0.115574 1.224115 -3.394710
H -1.631921 0.856515 -2.925265

TABLE SI.7: $^{3}MC_{1}$ coordinates at B3LYP*/3-21G/PCM(Acetonitrile)

53

N 2.011148 -1.157540 -0.558657
C 2.733695 -0.100797 -0.052888
C 4.101398 0.079720 -0.377194
C 4.709849 -0.872020 -1.229883
C 3.969415 -1.940021 -1.706490
C 2.616396 -2.059576 -1.353458
C 2.086286 0.817018 0.831529
C 2.808353 1.911217 1.369552
C 4.188228 2.083746 1.011092
C 4.809165 1.202402 0.173758
C 2.116278 2.781285 2.247385
C 0.786859 2.540369 2.551452
C 0.134266 1.432333 1.979410
N 0.773937 0.601548 1.145100
Rh -0.000026 -1.224616 -0.000040
N -0.773748 0.602089 -1.144715
C -2.086159 0.817400 -0.831376
C -2.808155 1.911710 -1.369278
C -2.115924 2.782040 -2.246735
C -0.786428 2.541284 -2.550554
C -0.133909 1.433132 -1.978635
C -2.733720 -0.100644 0.052727
C -4.101491 0.079770 0.376788
C -4.809196 1.202524 -0.174109
C -4.188122 2.084080 -1.011117
C -4.710075 -0.872112 1.229223
C -3.969698 -1.940154 1.705831
C -2.616614 -2.059620 1.353032
N -2.011253 -1.157477 0.558450
N 0.615286 -2.130286 1.927293
H -0.904904 1.218253 2.201794
H 0.237954 3.188033 3.223727
H 4.728054 2.928545 1.425192
H 2.637201 3.630582 2.677050
H 5.755334 -0.760039 -1.497011
H 5.853265 1.330599 -0.090568
H 2.024805 -2.892570 -1.709743
H 4.411113 -2.689618 -2.350857
H 0.905304 1.219135 -2.200896
H -0.237416 3.189155 -3.222544
H -4.727915 2.928939 -1.425142
H -2.636795 3.631422 -2.676297
H -5.755616 -0.760215 1.496159
H -5.853372 1.330596 0.089977
H -2.025066 -2.892657 1.709283
H -4.411487 -2.689843 2.350030
H 0.471942 -3.149705 1.848355
H 1.616186 -1.953052 2.089956
H 0.076591 -1.763919 2.724671
N -0.615680 -2.129911 -1.927278
H -0.077174 -1.763278 -2.724660
H -0.472200 -3.149333 -1.848643
H -1.616638 -1.952784 -2.089744

TABLE SI.8: $^3MC_2$ coordinates at B3LYP*/3-21G/PCM(Acetonitrile)

53

N -1.963897 0.623830 0.982750
C -2.625956 0.190657 -0.124665
C -3.885968 0.716820 -0.496684
C -4.449224 1.704377 0.340643
C -3.770762 2.112554 1.470931
C -2.518425 1.550154 1.760684
C -2.021552 -0.838737 -0.909666
C -2.676906 -1.314031 -2.069266
C -3.938647 -0.743052 -2.439661
C -4.521807 0.227900 -1.683720
C -2.052021 -2.347126 -2.799750
C -0.852657 -2.862609 -2.357354
C -0.266660 -2.346304 -1.194073
N -0.826268 -1.359257 -0.494578
Rh -0.012524 -0.506604 1.229034
N 0.787474 1.413131 0.005056
C 2.048473 1.230608 -0.466689
C 2.729740 2.213446 -1.223654
C 2.045316 3.419523 -1.486421
C 0.768228 3.591246 -0.998743
C 0.171510 2.560721 -0.252737
C 2.709395 -0.000202 -0.160641
C 4.034127 -0.231423 -0.602948
C 4.691975 0.778810 -1.377693
C 4.064551 1.949978 -1.676299
C 4.637304 -1.455515 -0.244183
C 3.936632 -2.370007 0.516103
C 2.625507 -2.070220 0.910176
N 2.037785 -0.921896 0.571550
N -1.329541 -1.766143 2.473294
H 0.670655 -2.743003 -0.826307
H -0.347837 -3.662825 -2.886331
H -4.426567 -1.111211 -3.336936
H -2.524235 -2.732754 -3.698377
H -5.415138 2.133090 0.090502
H -5.482599 0.646448 -1.967712
H -1.955793 1.865229 2.632976
H -4.179313 2.863535 2.137590
H -0.827720 2.687596 0.143688
H 0.211871 4.505330 -1.174360
H 4.570310 2.711885 -2.261934
H 2.531287 4.200620 -2.063725
H 5.652622 -1.667682 -0.566052
H 5.705559 0.591326 -1.718981
H 2.042604 -2.764957 1.508026
H 4.373845 -3.316039 0.814451
H -0.748737 -2.457480 2.949650
H -1.847896 -1.258019 3.187935
H -2.007638 -2.277250 1.910455
N 0.785761 0.415043 2.953303
H 1.096206 1.363870 2.746373
H 0.138764 0.477625 3.738657
H 1.601078 -0.096537 3.292144

TABLE SI.9: $^{3}MC_{3}$ coordinates at B3LYP*/3-21G/PCM(Acetonitrile)

53

N 2.080282 -1.010279 -0.763976
C 2.803368 -0.035908 -0.110037
C 4.171987 0.191031 -0.387437
C 4.787954 -0.628856 -1.362917
C 4.047028 -1.607553 -2.005770
C 2.690780 -1.777283 -1.688639
C 2.134048 0.752278 0.866844
C 2.829913 1.772702 1.558292
C 4.217721 1.991704 1.258490
C 4.862760 1.230521 0.325422
C 2.104653 2.522968 2.515090
C 0.767698 2.239558 2.741241
C 0.139194 1.208476 2.020293
N 0.810740 0.486265 1.109302
Rh 0.077435 -1.105552 -0.140322
N -0.896698 0.738982 -1.130425
C -2.213572 0.859190 -0.813261
C -3.014664 1.902815 -1.338264
C -2.391720 2.815450 -2.224528
C -1.050745 2.665961 -2.540472
C -0.316518 1.611571 -1.966316
C -2.778644 -0.105499 0.075285
C -4.144954 -0.006285 0.437531
C -4.936594 1.067596 -0.097746
C -4.396021 1.983575 -0.953766
C -4.667779 -0.987714 1.312190
C -3.845659 -2.002265 1.770673
C -2.501376 -2.043300 1.368169
N -1.973216 -1.114616 0.546670
N 0.667541 -2.235102 1.830007
H -0.903797 0.967732 2.184581
H 0.191541 2.797645 3.468609
H 4.742941 2.776410 1.792179
H 2.603620 3.314608 3.063864
H 5.836715 -0.484731 -1.599205
H 5.911206 1.397623 0.103075
H 2.102034 -2.536262 -2.185803
H 4.493574 -2.249134 -2.754825
H 0.735598 1.474662 -2.187045
H -0.554029 3.348415 -3.218888
H -5.000940 2.787815 -1.358932
H -2.972010 3.627913 -2.649118
H -5.709452 -0.938506 1.611370
H -5.979857 1.128913 0.192891
H -1.854452 -2.838758 1.710246
H -4.217054 -2.771865 2.435625
H 0.412825 -3.230179 1.749143
H 1.692307 -2.158240 1.887806
H 0.249069 -1.827288 2.676546
N -0.553164 -2.588548 -1.560828
H -0.391287 -2.257796 -2.525359
H -0.052652 -3.479617 -1.416227
H -1.562008 -2.760835 -1.443480

TABLE SI.10: ^{3}LC coordinates at B3LYP*/6-311G(d,f)/PCM(Acetonitrile)

53

N 1.929209 0.712291 -0.968539
C 2.697730 0.103442 -0.014147
C 4.045458 0.458517 0.210298
C 4.589175 1.481711 -0.596223
C 3.800510 2.085231 -1.553704
C 2.469087 1.676109 -1.716088
C 2.088405 -0.921869 0.765020
C 2.828215 -1.588484 1.765072
C 4.194837 -1.210878 1.977235
C 4.778456 -0.230186 1.231969
C 2.164777 -2.596884 2.497794
C 0.847430 -2.889854 2.212944
C 0.179962 -2.182979 1.201410
N 0.783822 -1.225258 0.498858
Rh -0.013319 -0.057751 -1.025247
N -0.798923 1.269514 0.366780
C -2.075478 1.019519 0.678325
C -2.808108 1.812427 1.618117
C -2.106302 2.902016 2.222943
C -0.789165 3.134068 1.881328
C -0.147584 2.311027 0.952767
C -2.704488 -0.116237 0.009973
C -4.069509 -0.448406 0.285715
C -4.768935 0.332342 1.200019
C -4.131498 1.477098 1.877517
C -4.611082 -1.574067 -0.412731
C -3.806077 -2.273727 -1.298314
C -2.487964 -1.888483 -1.508191
N -1.950233 -0.807219 -0.847691
N 0.685890 -1.461997 -2.442406
H -0.852841 -2.399110 0.958576
H 0.309310 -3.659452 2.754407
H 4.759188 -1.726220 2.748259
H 2.696327 -3.134397 3.277310
H 5.622313 1.785307 -0.457562
H 5.814576 0.047040 1.400474
H 1.837940 2.142868 -2.462004
H 4.185412 2.873917 -2.189810
H 0.881017 2.471477 0.662313
H -0.232618 3.952952 2.323536
H -4.713809 2.055730 2.585879
H -2.616569 3.534205 2.942351
H -5.641478 -1.867288 -0.241926
H -5.804829 0.105647 1.427270
H -1.840704 -2.422601 -2.188576
H -4.189807 -3.131686 -1.839729
H 0.070107 -1.583629 -3.246225
H 1.592386 -1.188539 -2.822941
H 0.819975 -2.385043 -2.028640
N -0.727783 1.180795 -2.580392
H -0.870050 2.141368 -2.267386
H -0.115574 1.224115 -3.394710
H -1.631921 0.856515 -2.925265

TABLE SI.11: $^3MC_1$ coordinates at B3LYP*/6-311G(d,f)/PCM(Acetonitrile)

53

N -2.018751 -1.182436 0.538960
C -2.784287 -0.140884 0.089132
C -4.156351 -0.033344 0.417501
C -4.722382 -1.049888 1.215532
C -3.939473 -2.106842 1.629653
C -2.586282 -2.142719 1.271112
C -2.178139 0.841249 -0.756773
C -2.946899 1.923445 -1.248826
C -4.326753 2.022916 -0.876682
C -4.906283 1.082619 -0.079937
C -2.295867 2.845532 -2.097422
C -0.966925 2.661053 -2.418674
C -0.278170 1.557798 -1.887803
N -0.870024 0.684801 -1.083235
Rh -0.000247 -1.182236 0.000636
N 0.871527 0.682337 1.086299
C 2.179067 0.839206 0.757834
C 2.948539 1.921022 1.249579
C 2.298770 2.842282 2.100034
C 0.970375 2.657341 2.423344
C 0.280833 1.554489 1.892657
C 2.783834 -0.142033 -0.090124
C 4.155391 -0.034296 -0.420476
C 4.906077 1.081270 0.076768
C 4.327782 2.020870 0.875226
C 4.720124 -1.050252 -1.220160
C 3.936500 -2.106826 -1.633935
C 2.583802 -2.142821 -1.273560
N 2.017435 -1.182956 -0.539955
N -0.567066 -1.969105 -1.964576
H 0.766554 1.384205 -2.125346
H -0.442432 3.347706 -3.073731
H -4.906547 2.861330 -1.250798
H -2.848821 3.691317 -2.495251
H -5.772124 -0.997392 1.488513
H -5.955357 1.158082 0.189119
H -1.958263 -2.969451 1.580204
H -4.344114 -2.913016 2.230615
H -0.763475 1.380488 2.131745
H 0.446924 3.343401 3.079854
H 4.908154 2.859019 1.249050
H 2.852235 3.687833 2.497639
H 5.769465 -0.997655 -1.494654
H 5.954723 1.157009 -0.193875
H 1.955170 -2.969205 -1.582349
H 4.340231 -2.912611 -2.236031
H -0.505347 -2.988261 -1.947472
H -1.528559 -1.738722 -2.210110
H 0.024882 -1.632088 -2.722706
N 0.565876 -1.973348 1.964481
H -0.024715 -1.635786 2.723420
H 0.501768 -2.992330 1.946206
H 1.528080 -1.745451 2.209507

TABLE SI.12: $^3MC_2$ coordinates at B3LYP*/6-311G(d,f)/PCM(Acetonitrile)

53

N -1.967216 0.651967 0.965636
C -2.618560 0.160690 -0.133483
C -3.880451 0.665395 -0.537106
C -4.454808 1.694974 0.247241
C -3.785824 2.166915 1.364649
C -2.532659 1.625206 1.701430
C -2.002602 -0.893739 -0.874460
C -2.650263 -1.422790 -2.018004
C -3.920288 -0.881978 -2.417342
C -4.513675 0.119424 -1.704865
C -2.010491 -2.477984 -2.711263
C -0.795872 -2.960082 -2.256221
C -0.206621 -2.394272 -1.114441
N -0.793599 -1.386668 -0.443862
Rh -0.002000 -0.460068 1.255875
N 0.757475 1.403867 -0.006075
C 2.024511 1.221817 -0.492146
C 2.677247 2.191035 -1.293345
C 1.971079 3.382264 -1.585623
C 0.694790 3.556622 -1.082223
C 0.113904 2.546620 -0.292935
C 2.706995 0.014257 -0.157504
C 4.027887 -0.215588 -0.617623
C 4.662398 0.778818 -1.437215
C 4.011590 1.933831 -1.762320
C 4.653609 -1.425670 -0.234109
C 3.974930 -2.330891 0.565951
C 2.664998 -2.039754 0.981686
N 2.056249 -0.895025 0.618414
N -1.337771 -1.675339 2.521857
H 0.742065 -2.756278 -0.743051
H -0.285163 -3.769533 -2.762370
H -4.399559 -1.294813 -3.298489
H -2.481166 -2.902257 -3.591812
H -5.418396 2.105957 -0.035063
H -5.475478 0.520300 -2.006591
H -1.983499 1.983913 2.563989
H -4.206105 2.950061 1.983105
H -0.879037 2.674771 0.112061
H 0.129053 4.458382 -1.281058
H 4.492470 2.684848 -2.380118
H 2.439123 4.147090 -2.196410
H 5.664451 -1.633988 -0.568655
H 5.671370 0.591435 -1.788859
H 2.107397 -2.726886 1.609646
H 4.432197 -3.261292 0.878466
H -0.737600 -2.305626 3.076644
H -1.913931 -1.107473 3.158185
H -1.955715 -2.242037 1.924304
N 0.782380 0.529273 2.956698
H 1.062022 1.483457 2.685167
H 0.112891 0.591536 3.737620
H 1.619029 0.024257 3.287861

TABLE SI.13: $^3MC_3$ coordinates at B3LYP*/6-311G(d,f)/PCM(Acetonitrile)

53

N 2.093730 -0.927084 -0.848789
C 2.833607 -0.012675 -0.144259
C 4.199687 0.219723 -0.412622
C 4.793385 -0.533403 -1.447752
C 4.034237 -1.451767 -2.147224
C 2.684094 -1.626139 -1.822918
C 2.175368 0.711285 0.885995
C 2.880418 1.670127 1.646994
C 4.266396 1.891761 1.357577
C 4.899299 1.195624 0.370847
C 2.161737 2.351138 2.653158
C 0.827734 2.060223 2.853577
C 0.198698 1.091473 2.056473
N 0.855126 0.437772 1.101287
Rh 0.103740 -1.046564 -0.205389
N -0.974008 0.813202 -1.106975
C -2.278473 0.890743 -0.762033
C -3.119559 1.924385 -1.236818
C -2.540152 2.878744 -2.101625
C -1.207555 2.773208 -2.445765
C -0.442863 1.719606 -1.918205
C -2.797180 -0.120717 0.106284
C -4.158480 -0.076345 0.490407
C -4.987540 0.988304 0.004824
C -4.491616 1.947235 -0.825725
C -4.634829 -1.102969 1.332383
C -3.775639 -2.103007 1.735147
C -2.441332 -2.077786 1.308007
N -1.955638 -1.112244 0.525119
N 0.717031 -2.360642 1.710377
H -0.845892 0.841287 2.200708
H 0.248738 2.563949 3.619120
H 4.806031 2.628971 1.943958
H 2.662843 3.096807 3.262899
H 5.842157 -0.387219 -1.687584
H 5.950082 1.370196 0.161145
H 2.077344 -2.343599 -2.360610
H 4.461241 -2.045475 -2.947182
H 0.608787 1.612709 -2.164400
H -0.737055 3.488213 -3.111482
H -5.131337 2.744274 -1.192768
H -3.148520 3.690586 -2.489292
H -5.673543 -1.101110 1.649123
H -6.028970 1.009504 0.311305
H -1.761365 -2.866203 1.602569
H -4.106764 -2.913606 2.374194
H 0.533826 -3.352870 1.567327
H 1.725301 -2.262361 1.817629
H 0.296871 -2.084498 2.596088
N -0.568790 -2.503499 -1.604743
H -0.492630 -2.166931 -2.565706
H -0.053439 -3.382422 -1.544544
H -1.550824 -2.730003 -1.454281

TABLE SI.14: $^3MC_4$ coordinates at B3LYP*/6-311G(d,f)/PCM(Acetonitrile)

53

N 2.229786 -0.937251 0.825447
C 2.933783 -0.150535 -0.026062
C 4.306353 -0.376154 -0.306161
C 4.932673 -1.458958 0.352416
C 4.202431 -2.243044 1.218191
C 2.841971 -1.949872 1.427311
C 2.249584 0.935591 -0.667135
C 2.945193 1.756466 -1.587464
C 4.329137 1.502457 -1.848132
C 4.985294 0.478475 -1.228730
C 2.229244 2.795188 -2.219405
C 0.888474 2.976208 -1.937563
C 0.265553 2.129306 -1.019827
N 0.926940 1.146397 -0.390628
Rh 0.000983 -0.042572 1.055393
N -0.942728 -1.155562 -0.433218
C -2.259381 -0.900302 -0.702511
C -2.970052 -1.657944 -1.664425
C -2.275890 -2.681249 -2.343851
C -0.942270 -2.910271 -2.064019
C -0.302870 -2.124354 -1.103592
C -2.922555 0.163489 -0.005085
C -4.289122 0.432416 -0.274144
C -4.983439 -0.357272 -1.242731
C -4.347789 -1.360633 -1.913791
C -4.894662 1.489614 0.442289
C -4.151981 2.207471 1.354042
C -2.799322 1.873818 1.551203
N -2.205425 0.886252 0.892534
N 0.925291 1.100786 2.609905
H -0.781579 2.245270 -0.770178
H 0.309410 3.760621 -2.410814
H 4.847494 2.142684 -2.555187
H 2.737382 3.441763 -2.928644
H 5.982885 -1.664299 0.166914
H 6.035764 0.294830 -1.433394
H 2.240708 -2.559631 2.094925
H 4.651485 -3.083747 1.735530
H 0.740379 -2.276459 -0.856407
H -0.380524 -3.685736 -2.571716
H -4.877934 -1.952791 -2.653244
H -2.795833 -3.279449 -3.086256
H -5.939492 1.727434 0.265584
H -6.029453 -0.140866 -1.438227
H -2.189237 2.429973 2.256308
H -4.585974 3.025994 1.917705
H 1.773105 0.646993 2.948070
H 1.198426 2.023080 2.269191
H 0.324373 1.259789 3.418065
N -0.915102 -1.248641 2.568549
H -1.204441 -2.150611 2.188594
H -0.301887 -1.450743 3.357510
H -1.752796 -0.801688 2.939938

TABLE SI.15: ^{3}LC frequency list (cm^{-1}) at B3LYP*/3-21G/PCM(Acetonitrile)

26.5870	43.7821	50.8861
84.2061	95.2964	119.7426
154.8413	169.9181	177.5552
185.8561	194.1693	196.5582
213.4814	218.2601	230.6529
242.2956	268.6582	269.2842
281.8737	286.2696	292.9050
295.9072	319.0426	329.7629
341.7281	346.1606	433.3961
444.4182	449.1437	453.2036
457.2686	472.8662	479.2470
495.8176	503.5634	514.0445
523.8174	526.5861	530.5916
546.5028	570.8359	574.5428
583.2235	584.9817	651.0387
663.1359	687.8511	710.0911
722.5622	736.4603	751.8536
756.1847	762.1432	765.1098
770.2944	776.1211	801.2493
809.3609	818.1958	824.0507
830.1644	832.1637	849.4079
880.1567	885.5176	890.7776
897.9295	908.5386	913.5933
937.6512	939.5865	950.0456
962.6167	989.8469	992.6616
997.3235	1020.3649	1031.4389
1042.5084	1056.5025	1060.4160
1061.8967	1072.2548	1083.5203
1085.4915	1115.6529	1120.4466
1123.6591	1134.2827	1154.1207
1199.3942	1206.0441	1218.9463
1234.4095	1239.2950	1245.9263
1248.8989	1255.4994	1294.5227
1303.4556	1315.6587	1323.5768
1330.8703	1354.4263	1373.4417
1375.2453	1410.3154	1418.2342
1421.7289	1433.3538	1441.2631
1443.0168	1453.3398	1465.2330
1471.6724	1487.7384	1502.3679
1505.0294	1518.1906	1521.7872
1529.7348	1533.6459	1563.8490
1576.0551	1594.0207	1600.3326
1638.1116	1722.2284	1740.0540
1747.6324	1764.2698	3201.8654
3207.7573	3211.2248	3212.2261
3213.5070	3215.3131	3217.1225
3224.9218	3226.5755	3228.6642
3230.5561	3232.1096	3246.0251
3247.2419	3263.3616	3266.6681
3344.7645	3348.8764	3441.3418
3441.5257	3446.0703	3454.7555

TABLE SI.16: $^{3}MC_{1}$ frequency list (cm^{-1}) at B3LYP*/3-21G/PCM(Acetonitrile)

26.4836	35.3636	49.3705
85.4921	95.5300	104.6455
114.6799	143.4658	155.7092
160.3916	165.0186	175.2455
183.5679	191.0179	199.8552
219.9198	239.5122	243.5848
245.8885	250.5305	255.1320
266.7583	284.7748	293.4307
311.2784	408.4563	435.7973
442.0792	443.6050	448.3272
470.5336	480.0375	489.8097
502.5018	503.4074	529.5336
531.4824	536.2473	539.2597
579.9622	581.5021	585.1527
588.4854	641.6100	646.4374
670.2042	680.5841	728.4631
738.7379	748.9102	750.1473
758.5718	760.4900	776.4475
778.2101	800.6008	801.0717
805.0265	831.0161	833.2284
846.2657	894.4990	896.5911
897.5190	898.3981	922.9835
931.9116	962.4615	964.8333
992.2439	997.0561	998.5219
1002.9002	1042.8714	1043.9809
1056.4475	1058.1401	1062.5708
1063.7812	1070.1139	1071.1393
1079.3531	1081.6893	1118.3113
1120.1580	1127.4290	1130.0118
1192.9369	1195.5991	1206.6295
1207.0189	1232.5923	1233.1079
1246.9469	1249.5603	1254.1615
1259.4206	1287.2369	1292.7321
1313.7225	1321.6175	1322.0091
1327.3257	1328.9537	1370.6340
1373.5328	1384.2660	1433.7575
1435.8957	1440.6732	1441.6113
1474.4091	1475.2350	1486.0622
1487.8990	1519.2337	1520.4943
1526.0045	1526.9673	1563.5771
1567.1252	1592.8754	1593.5958
1599.9135	1602.0030	1635.7496
1636.5430	1703.7071	1715.6740
1724.6106	1735.2080	3198.2322
3199.4942	3202.6695	3205.9582
3206.6340	3206.9085	3208.3626
3212.1349	3213.4327	3219.8605
3230.6929	3232.3948	3234.0092
3236.1452	3250.3835	3260.6984
3347.6205	3350.5886	3446.3031
3456.2301	3457.3642	3475.6423

TABLE SI.17: $^3MC_2$ frequency list (cm^{-1}) at B3LYP*/3-21G/PCM(Acetonitrile)

30.9216	40.0417	40.0866
77.7618	85.3738	111.9041
116.2205	122.6862	153.3480
163.1738	171.4744	173.5922
177.6246	181.3259	194.8699
201.0455	209.5145	231.4141
247.5909	248.8251	262.8715
278.7208	288.3601	303.9465
303.9779	383.8486	387.4643
442.0409	443.0489	443.5601
450.2433	478.6720	481.0230
502.2781	504.8991	528.3674
528.7835	538.7423	539.7845
579.6579	580.0333	586.5119
587.0466	645.6962	648.2409
675.7196	682.0392	710.0542
723.7787	743.9629	744.2621
751.7978	754.0473	760.2815
765.4031	774.5700	777.5656
802.0593	802.2715	830.9160
831.3268	893.9262	896.6872
897.3844	898.0025	929.7386
934.1635	963.4868	963.9529
989.9675	990.1945	998.4517
998.9640	1042.5365	1042.6850
1056.5402	1056.6120	1061.7953
1061.8152	1067.8158	1069.3737
1080.0370	1082.3737	1121.3375
1122.5148	1128.4000	1131.5191
1195.7791	1196.4041	1206.2433
1206.2576	1232.0848	1232.5131
1247.5886	1249.9062	1255.0482
1255.5011	1294.6711	1296.3965
1310.0482	1312.1929	1322.1641
1322.1773	1329.0037	1329.8372
1373.2507	1374.2007	1432.4093
1433.0013	1441.3262	1443.7307
1474.8390	1475.0701	1486.5964
1488.1373	1520.8901	1522.1861
1525.2557	1526.2355	1567.6564
1567.8501	1594.2117	1594.2593
1600.7201	1600.7576	1634.8788
1635.7170	1709.2404	1709.7405
1713.0283	1713.6496	3200.0832
3200.0927	3205.1833	3205.1916
3208.0232	3208.0436	3211.9774
3212.0285	3213.9094	3213.9366
3232.8778	3232.9035	3233.4788
3233.5194	3250.1677	3250.4103
3343.0719	3344.7207	3451.8903
3452.2202	3470.0538	3470.1147

TABLE SI.18: $^{3}MC_3$ frequency list (cm^{-1}) at B3LYP*/3-21G/PCM(Acetonitrile)

13.9077	31.5232	34.6632
40.5337	71.0909	81.0332
109.5365	113.5509	141.3376
147.3739	153.1249	168.1089
175.4569	188.4624	189.4869
194.7070	210.2970	227.5994
243.1499	244.3219	265.7536
277.6276	285.1117	297.8522
306.7830	335.8196	436.3159
437.2203	444.6380	448.4074
458.4285	475.8384	480.1979
501.5597	507.5233	524.2150
527.3438	533.6463	541.1701
579.7294	580.0064	582.3622
582.6298	627.2377	643.2797
646.9939	674.7207	675.3935
682.0578	745.5381	752.7329
755.2874	757.8575	771.7786
772.8162	782.1628	795.3374
801.3943	801.8557	827.5553
829.8942	893.1305	896.8008
897.0540	902.7870	922.8386
929.6099	960.9034	964.6786
989.5667	991.5301	991.8662
995.1745	1041.7652	1042.6138
1055.2770	1057.0237	1061.4982
1062.3491	1067.1261	1068.2180
1079.3197	1080.6341	1117.5387
1120.1784	1126.1107	1128.6745
1192.4566	1195.1078	1205.0892
1206.2124	1231.8320	1232.5309
1235.5961	1243.5637	1248.8113
1252.1864	1258.2052	1290.2996
1291.7623	1319.3571	1321.7380
1328.5762	1328.8265	1365.3488
1371.2929	1373.8018	1434.8448
1437.2205	1440.3584	1443.8089
1469.6173	1472.1904	1485.3234
1487.5997	1519.0216	1520.7759
1523.5735	1525.8590	1564.0040
1568.2281	1589.9383	1592.4068
1596.8570	1600.9282	1634.2098
1635.6602	1703.8409	1709.0800
1717.4486	1722.8257	3198.8607
3200.5870	3206.7795	3207.3593
3207.7471	3210.1740	3212.6357
3213.3018	3214.3139	3224.1949
3232.6851	3232.7024	3233.6418
3237.4129	3247.8583	3258.7965
3338.5240	3351.0143	3437.6815
3457.6574	3469.1371	3481.1155

TABLE SI.19: ^{3}LC frequency list (cm^{-1}) at B3LYP*/6-311G(d,f)/PCM(Acetonitrile)

26.3764	43.6358	50.5603
81.6771	94.2050	119.8276
148.1978	167.2367	174.2062
188.1524	196.6708	198.4407
201.2853	214.9625	228.7016
234.3049	267.2738	271.1057
275.0846	284.3190	290.2241
295.0877	320.2215	325.1137
338.1916	343.1434	417.0880
429.5496	435.2366	438.7987
440.7983	454.4310	461.4697
475.1973	481.6479	498.6029
513.0742	514.2532	518.1225
528.4456	544.1091	553.8333
558.7874	569.5843	620.0198
639.9038	665.1149	684.5081
692.3880	710.5373	726.0289
734.5247	735.5102	741.8931
752.2853	774.9623	777.9344
781.6835	784.7343	798.9458
804.2388	808.3144	821.0999
835.6772	845.6539	853.2381
855.2845	874.8042	881.4957
890.1451	892.6936	922.6065
931.9464	960.6922	963.5773
971.8263	985.4518	986.7201
1008.7437	1010.7888	1011.2279
1038.6497	1050.3940	1085.5561
1086.5193	1113.8083	1115.5210
1116.4122	1128.5286	1133.7218
1171.1755	1174.1612	1194.1872
1217.6725	1233.0827	1234.7525
1237.7851	1246.8118	1282.6241
1296.8836	1311.7841	1342.6226
1345.8303	1367.0716	1369.6566
1401.4925	1413.5315	1422.7760
1426.1086	1439.1199	1441.0524
1445.5140	1447.7125	1454.4593
1457.4425	1479.9104	1506.2260
1518.6203	1523.1171	1536.2864
1549.7271	1555.0712	1592.3658
1609.7984	1616.6728	1625.6857
1653.8263	1711.2796	1720.0687
1732.8644	1744.7202	3178.2172
3184.8824	3187.9685	3188.4440
3190.0620	3191.3281	3194.4502
3201.1945	3203.3287	3204.0876
3204.9464	3206.7336	3222.8018
3223.5708	3238.2192	3242.7409
3445.5280	3448.2595	3529.2684
3530.8842	3537.2206	3543.2404

TABLE SI.20: $^{3}MC_{1}$ frequency list (cm^{-1}) at B3LYP*/6-311G(d,f)/PCM(Acetonitrile)

25.7061	39.9274	46.8327
78.2215	91.9508	99.1697
108.6359	143.0848	150.5680
153.1790	162.7209	166.7768
173.2297	194.4876	215.2114
221.4561	233.5507	241.6340
243.0868	249.4429	256.2513
263.3892	279.1691	302.7424
319.2012	378.6722	419.2373
426.0212	429.9164	433.6637
446.9356	457.1024	466.3977
489.7874	491.3928	514.0484
516.6864	520.4321	521.1805
551.8774	554.5707	564.8525
566.1687	608.1331	613.6854
646.1927	657.2251	698.5162
718.6228	727.7779	730.4276
731.7663	733.9146	743.4658
744.7402	777.4027	778.9028
796.7554	800.3459	801.2916
838.0710	839.5496	845.4941
853.2393	855.5199	875.4400
880.7947	911.7669	923.0178
959.5181	964.0983	973.3821
974.9644	984.6245	987.1707
1006.8832	1008.4819	1009.4333
1011.8975	1046.6264	1047.8771
1081.7110	1084.1319	1113.1957
1115.5469	1123.6612	1125.7995
1164.3718	1167.7901	1172.4928
1174.5473	1233.1598	1233.5442
1234.8986	1237.7762	1246.4370
1248.0411	1279.4864	1284.9094
1329.7150	1339.2484	1339.7062
1343.3057	1346.7565	1366.8283
1371.0842	1408.6485	1435.6718
1437.3228	1443.5540	1444.5297
1453.0214	1455.1627	1478.6698
1479.8003	1520.9564	1522.7050
1544.8674	1547.4769	1596.0551
1601.4595	1616.1351	1616.5479
1624.7237	1627.6266	1648.5920
1651.3002	1689.1237	1692.9853
1710.3247	1722.8238	3173.8488
3177.1257	3178.9866	3181.8782
3182.5193	3184.2725	3184.5780
3187.5947	3190.5117	3196.6208
3200.6803	3205.8998	3206.5077
3209.3863	3220.1038	3227.1244
3448.0091	3450.7791	3537.9936
3544.1928	3547.3047	3562.8457

TABLE SI.21: $^{3}MC_{2}$ frequency list (cm^{-1}) at B3LYP*/6-311G(d,f)/PCM(Acetonitrile)

22.8085	30.2209	35.0744
58.2039	80.3200	98.6499
102.6451	104.7054	135.9038
143.6194	163.8655	176.8111
180.0086	193.3287	207.8030
214.9004	223.3974	228.1855
233.9106	242.4705	259.1788
273.3071	277.7469	302.8642
309.2638	359.4920	367.4267
424.4099	430.3568	430.5676
433.0659	456.1661	458.8719
488.5626	494.6925	516.2092
517.4868	517.9722	519.3538
550.0486	550.5748	564.5320
565.0714	613.7889	614.9509
652.5574	657.4101	706.6507
718.9747	725.8536	727.5197
731.2375	731.4760	733.7254
741.5622	747.0359	748.7437
776.6860	776.8227	796.8486
797.2755	837.3607	838.0139
853.2826	853.4046	875.2677
878.7457	919.4402	923.7149
959.0834	959.1197	962.6899
963.1839	984.9276	984.9692
1006.4392	1006.5411	1009.2228
1009.2418	1043.6365	1046.0647
1079.8950	1081.5728	1113.6270
1114.2474	1122.5248	1124.9320
1166.4299	1166.5991	1173.6930
1173.7180	1230.6584	1233.0132
1233.1599	1234.1006	1245.8945
1246.4953	1282.0272	1283.7176
1337.3307	1337.6552	1338.6533
1341.7587	1344.5763	1344.8866
1367.3679	1368.0894	1434.6419
1434.9844	1441.1956	1442.4796
1452.2448	1453.8399	1476.4722
1478.6840	1522.0756	1523.0793
1542.0539	1543.9447	1598.8627
1599.2368	1617.0175	1617.3310
1626.3362	1626.4483	1648.9949
1649.7422	1694.6516	1695.1404
1699.1984	1699.6309	3175.6142
3175.6207	3183.2217	3183.2649
3184.2236	3184.2411	3188.9996
3189.0369	3189.5048	3189.6268
3204.8464	3204.9509	3205.2397
3205.3193	3218.3581	3218.6693
3445.7415	3446.9403	3541.5499
3541.8494	3558.0438	3558.1203

TABLE SI.22: $^3MC_3$ frequency list (cm^{-1}) at B3LYP*/6-311G(d,f)/PCM(Acetonitrile)

22.4009	31.5745	33.0783
72.9273	78.7283	92.5574
107.0122	118.7688	136.0399
137.8223	148.2658	158.4326
163.5686	169.2674	185.9853
194.7923	210.1479	221.6593
232.1655	232.8810	261.6196
266.5266	276.2561	280.0516
298.2712	318.1791	418.2310
424.7482	427.9954	434.5449
443.3415	451.6130	459.4627
488.0414	497.0532	511.1681
512.5084	513.9343	519.5083
547.1179	550.0889	564.3466
565.8368	591.0816	609.5805
613.2940	638.1369	649.1070
661.2477	724.5056	725.5524
731.4008	732.9567	739.5958
747.9281	775.8632	776.8059
783.1472	787.7703	797.7599
799.6188	836.9576	841.8710
853.3222	853.5670	874.6995
887.2300	910.6187	922.6304
960.2157	961.1722	965.2152
970.2258	984.1721	985.1304
1005.2092	1009.8162	1010.5889
1012.6362	1044.0019	1045.1450
1077.6690	1079.9324	1106.1613
1110.8712	1118.3880	1121.0638
1162.8278	1167.0412	1171.7021
1172.9775	1225.7528	1228.0160
1232.6081	1236.3507	1243.1650
1246.4504	1262.2633	1276.5976
1279.2568	1335.1735	1338.2658
1345.6642	1347.4745	1365.0542
1367.5656	1384.8595	1434.5940
1435.4516	1440.2552	1444.4745
1450.3433	1451.0821	1475.7981
1477.7936	1520.4316	1520.7279
1540.5595	1544.6669	1594.4897
1598.9374	1609.0523	1614.3342
1621.4169	1626.0599	1647.1861
1650.0638	1687.7659	1697.0114
1702.2294	1704.7508	3173.4681
3177.3511	3180.5643	3183.4941
3186.9454	3186.9923	3187.5028
3187.5656	3191.0853	3204.2281
3204.7027	3205.2790	3206.8254
3215.7673	3220.6918	3229.8653
3443.2190	3451.0386	3533.7107
3543.2390	3555.6641	3562.6451

TABLE SI.23: $^3MC_4$ frequency list (cm^{-1}) at B3LYP*/6-311G(d,f)/PCM(Acetonitrile)

23.6268	29.2211	43.5883
80.4464	89.1625	101.8674
110.8693	123.5255	134.6011
152.0904	156.2791	164.6491
167.0374	176.5331	199.5561
209.2942	216.9810	232.1729
233.6681	235.3497	256.8910
262.2920	264.0508	299.0434
306.0938	396.3848	417.0174
420.7595	425.3770	428.1486
442.9462	446.0912	453.9536
483.6223	486.7896	511.9545
513.8449	514.9610	515.1849
545.0985	548.0822	559.4812
561.5146	609.2303	615.4299
653.3160	654.4369	725.9208
729.9168	730.3834	731.4268
737.6280	740.8491	751.2652
756.7627	778.6266	778.7972
801.1904	803.3084	811.1792
837.5224	838.5797	842.1715
855.9647	856.6603	874.8025
876.8968	913.7253	915.5285
968.0171	969.2485	973.1667
973.3142	984.7040	985.3441
1009.2687	1009.7599	1011.7147
1011.7342	1042.1242	1044.6400
1072.3383	1077.1630	1109.5091
1110.1669	1116.3347	1120.4311
1163.2537	1163.7386	1170.1557
1171.2687	1223.6333	1227.9645
1233.3385	1233.6068	1245.2494
1246.2182	1274.7626	1277.6558
1317.3024	1326.7892	1344.0361
1346.0428	1362.1622	1365.7190
1375.5901	1411.0204	1420.4262
1426.4457	1440.2395	1442.1173
1448.0483	1451.6712	1468.0150
1471.5225	1520.2632	1521.3225
1534.8436	1537.5720	1583.9442
1590.0539	1595.2798	1597.6876
1613.5067	1618.8585	1625.3036
1626.9859	1692.2299	1698.1701
1713.4871	1718.1202	3175.0681
3175.1297	3178.8600	3180.6159
3182.7526	3185.1956	3185.9965
3187.6553	3188.8207	3189.1558
3203.3509	3204.2874	3206.6842
3207.5469	3220.2304	3223.9379
3447.0392	3450.3608	3537.6222
3538.9743	3545.1696	3549.9133
